# Gene editing of the E3 ligase *PIRE1* fine-tunes reactive oxygen species production for enhanced bacterial disease resistance in tomato

**DOI:** 10.1093/plcell/koaf049

**Published:** 2025-05-30

**Authors:** Bardo Castro, Suji Baik, Megann Tran, Jie Zhu, Tianrun Li, Andrea Tang, Nathalie Aoun, Alison C Blundell, Michael Gomez, Elaine Zhang, Myeong-Je Cho, Tiffany Lowe-Power, Shahid Siddique, Brian Staskawicz, Gitta Coaker

**Affiliations:** Department of Plant Pathology, University of California, Davis, Davis, CA 95616, USA; Department of Entomology and Nematology, University of California, Davis, Davis, CA 95616, USA; Department of Plant Pathology, University of California, Davis, Davis, CA 95616, USA; Department of Plant Pathology, University of California, Davis, Davis, CA 95616, USA; Department of Plant Pathology, University of California, Davis, Davis, CA 95616, USA; Department of Plant Pathology, University of California, Davis, Davis, CA 95616, USA; Department of Plant Pathology, University of California, Davis, Davis, CA 95616, USA; Department of Plant Pathology, University of California, Davis, Davis, CA 95616, USA; Department of Plant Pathology, University of California, Davis, Davis, CA 95616, USA; Department of Plant and Microbial Biology, University of California, Berkeley, CA 94720, USA; Innovative Genomics Institute, University of California, Berkeley, CA 94720, USA; Innovative Genomics Institute, University of California, Berkeley, CA 94720, USA; Department of Plant Pathology, University of California, Davis, Davis, CA 95616, USA; Department of Entomology and Nematology, University of California, Davis, Davis, CA 95616, USA; Department of Plant and Microbial Biology, University of California, Berkeley, CA 94720, USA; Innovative Genomics Institute, University of California, Berkeley, CA 94720, USA; Department of Plant Pathology, University of California, Davis, Davis, CA 95616, USA

## Abstract

Reactive oxygen species (ROS) accumulation is required for effective plant defense. Accumulation of the Arabidopsis (*Arabidopsis thaliana*) NADPH oxidase respiratory burst oxidase homolog D (RBOHD) is regulated by phosphorylation of a conserved C-terminal residue (T912) leading to ubiquitination by the RING E3 ligase Pbl13-interacting RING domain E3 ligase (PIRE). Arabidopsis *PIRE* knockouts exhibit enhanced ROS production and resistance to the foliar pathogen *Pseudomonas syringae*. Here, we identified 170 *PIRE* homologs, which emerged in tracheophytes and expanded in angiosperms. We investigated the role of tomato (*Solanum lycopersicum*) PIRE homologs in regulating ROS production, RBOH stability, and disease resistance. Mutational analyses of residues corresponding to T912 in the tomato RBOHD ortholog, SlRBOHB, affected protein accumulation and ROS production in a *PIRE-*dependent manner. Using genome editing, we generated mutants in 2 *S. lycopersicum PIRE* (*SlPIRE*) homologs. *SlPIRE1* edited lines (*Slpire1*) in the tomato cultivar M82 displayed enhanced ROS production upon treatment with flg22, an immunogenic epitope of flagellin. Furthermore*, Slpire1* exhibited decreased disease symptoms and bacterial accumulation when inoculated with foliar bacterial pathogens *P. syringae* and *Xanthomonas campestris*. However, *Slpire1* exhibited similar levels of colonization as wild type upon inoculation with diverse soil-borne pathogens. These results indicate that PIRE regulates RBOHs in multiple plant species and is a promising target for foliar disease control. This study also highlights the pathogen-specific role of *PIRE*, indicating its potential for targeted manipulation to enhance foliar disease resistance without affecting root-associated pathogenic interactions.

## Teaser

An E3 ligase helps control plant immune responses and regulates reactive oxygen species production, offering a strategy to boost disease resistance in leaves without affecting root-associated pathogens.

## Introduction

Crop production is impacted by diverse plant pathogens. Among 5 major food crops (potato, soybean, wheat, maize, and rice), losses due to pests and pathogens range between 17% and 30% globally (Savary et al. 2019). Plants contain innate immune receptors that can recognize all pathogen classes. Pathogen recognition can occur extracellularly via cell surface–localized pattern recognition receptors (PRRs) leading to pattern-triggered immunity (PTI) or intracellularly through recognition of pathogen-encoded effectors by nucleotide-binding domain leucine-rich repeat receptors (NLRs) leading to effector-triggered immunity (Li et al. 2024; Yuan et al. 2023). NLRs and PRRs mutually potentiate each other, and their activation leads to convergent responses (Yuan et al. 2023). Common plant immune responses include ion influxes, rapid production of reactive oxygen species (ROS), transcriptional reprogramming, deposition of structural barriers, and stomatal closure, all of which culminate in resistance (Yuan et al. 2023).

Much of our understanding of PTI comes from the conserved PRR, FLAGELLIN-SENSING 2 (FLS2). FLS2 is a leucine-rich repeat receptor kinase that perceives a 22 amino acid immunogenic epitope, flg22, from the bacterial flagellin C protein FliC (Zipfel et al. 2004). The molecular interaction between flg22 and FLS2 leads to recruitment of a somatic embryogenesis receptor kinase coreceptor (Chinchilla et al. 2007; Heese et al. 2007; Sun et al. 2013). In Arabidopsis (*Arabidopsis thaliana*), formation of the FLS2 receptor complex induces transphosphorylation of multiple intracellular kinases, including receptor-like cytoplasmic kinases, calcium-dependent protein kinases, and mitogen-activated protein kinases, which lead to multiple defense outputs (Couto and Zipfel 2016). To rapidly respond to pathogens, plant immune receptors and key signaling proteins are presynthesized and regulated through posttranslational modifications (PTMs). PTMs can affect all aspects of protein function including dynamic control or protein abundance, activity, and localization (Csizmok and Forman-Kay 2018; Lee et al. 2023). For instance, the FLS2 receptor complex and calcium and ROS production are regulated through multiple transphosphorylation events (Kadota et al. 2014; Li et al. 2014; Couto and Zipfel 2016; Zhang et al. 2018; Tian et al. 2019; Thor et al. 2020). Another key layer of posttranslational regulation is ubiquitination and subsequent degradation. For example, after the FLS2-flg22 immune complex forms, it is ubiquitinated by 2 U-box E3 ubiquitin ligases, PUB12 and PUB13, leading to its degradation and immune signal turnover (Lu et al. 2011).

One pivotal process regulated by phosphorylation is the production of apoplastic ROS by membrane-localized NADPH oxidases, termed respiratory burst oxidase homologs (RBOHs) in plants (Dubiella et al. 2013; Kadota et al. 2014; Li et al. 2014; Kimura et al. 2020; Lee et al. 2020; Castro et al. 2021). RBOHs produce superoxide (O^2•−^), which can be converted to hydrogen peroxide (H_2_O_2_), which is the most stable form and considered a key signaling molecule (Castro et al. 2021). RBOH activation during PTI leads to rapid and dynamic generation of ROS (Torres et al. 2002; Nühse et al. 2007; Zhang et al. 2007). Extracellular accumulation of ROS is involved in numerous processes including cell wall lignification, stomatal closure, and systemic acquired resistance (Melotto et al. 2006; Li et al. 2014; Kadota et al. 2015; Waszczak et al. 2018). Although de novo ROS production is crucial for defense, continual accumulation of hydrogen peroxide, superoxide, and hydroxyl radicals can lead to cellular oxidative damage (Kerchev and Van Breusegem 2022).

The production of ROS is essential for a robust immune response; however, this production must be dynamically regulated to minimize detrimental effects to the host. During pathogen perception, different kinase families phosphorylate N-terminal residues on Arabidopsis respiratory burst oxidase homolog D (RBOHD), leading to functional activation (Kadota et al. 2015; Zhang et al. 2018; Bender and Zipfel 2023). In recent years, research has shown that modification of C-terminal residues of Arabidopsis RBOHD (AtRBOHD) is also important for its regulation (Kimura et al. 2020; Lee et al. 2020). Our previous work identified the receptor-like cytoplasmic kinase PBS1-like kinase 13 (PBL13) that phosphorylates multiple AtRBOHD C-terminal residues to negatively regulate ROS production (Lee et al. 2020). PBL13 phosphorylates T912, which reduces AtRBOHD stability, and S862, which impacts enzyme activity (Lee et al. 2020). Crosstalk between phosphorylation and ubiquitination is critical to dynamically control protein levels (Lu et al. 2011; Swaney et al. 2013; Castro et al. 2021). The PBL13-interacting RING domain E3 ligase (PIRE) ubiquitinates AtRBOHD's C-terminus in a phosphorylation-dependent manner (Lee et al. 2020). Consistent with these results, *pbl13* and *pire* knockouts displayed enhanced AtRBOHD accumulation, immune-induced ROS production, and resistance to the bacterial pathogen *Pseudomonas syringae* (Lee et al. 2020). Upon pathogen perception, phosphatidic acid binds to RBOHD, inhibiting its interaction with PIRE (Qi et al. 2024).This suppression prevents RBOHD protein degradation, resulting in increased levels of RBOHD in the plasma membrane during pathogen perception (Qi et al. 2024).

Analysis of 112 plant RBOH homologs revealed high conservation of residue T912, which is important for PBL13-PIRE regulation (Castro et al. 2021). However, PBL13 is only found in the *Brassicaceae* (Lee et al. 2020). The conservation of RBOHD T912 indicates other plants may regulate RBOHs in a similar manner, but through different kinases, which can be exploited for disease control. In this manuscript, we have identified homologs of the Arabidopsis ubiquitin E3 ligase PIRE across the plant kingdom and investigated the importance of RBOH modification in the Solanaceae. We investigated the importance of *Solanum lycopersicum* (tomato) and Nicotiana *PIRE* homologs for regulation of ROS activity, utilizing the *S. lycopersicum* ortholog of AtRBOHD, SlRBOHB (Li et al. 2015). SlRBOHB abundance is also regulated at similar residues, and silencing in *N. benthamiana* implicated *PIRE* homologs in regulating RBOH abundance. Furthermore, we utilized clustered regularly interspaced short palindromic repeats (CRISPR) and CRISPR-associated protein 9-(cas9) to generate *S. lycopersicum pire* mutants. The *S. lycopersicum pire1* mutant exhibited higher ROS production upon immune activation and increased disease resistance to foliar bacterial pathogens. Our results provide evidence that crosstalk between phosphorylation and ubiquitination functions as a conserved regulatory module for plant RBOHs, and *PIRE* is a promising target to enhance disease resistance.

## Results

### Homologs of the E3 ubiquitin ligase PIRE are broadly conserved

Analysis of 112 RBOH homologs in plants revealed that the residue corresponding to T912 is highly conserved, indicating that PIRE-mediated regulation of ROS may also be conserved (Castro et al. 2021). Therefore, we first sought to identify PIRE homologs in various plant lineages. Previously, Arabidopsis RING domain proteins were classified into 8 different classes based on their metal ligand residues (Stone et al. 2005). While there are more than 470 RING E3 ligases in Arabidopsis, there are only 10 identified zinc-binding RING-C2s (Deshaies and Joazeiro 2009; Duplan and Rivas 2014; Metzger et al. 2014; Cho et al. 2017). AtPIRE is 319 amino acids (aa) in length and contains a modified RING-C2 domain on its C-terminal region from aa 244 to 290. The modified RING-C2 domain in Arabidopsis contains variable regions between the specific ligand binding sites (Supplementary Fig. S1). Utilizing Simple Modular Architecture Research Tool (SMART) (Letunic et al. 2021), we identified a low complexity region containing serine (S) and glutamic acid (D) repeats from aa 117 to 159 in AtPIRE.

**Figure 1.**
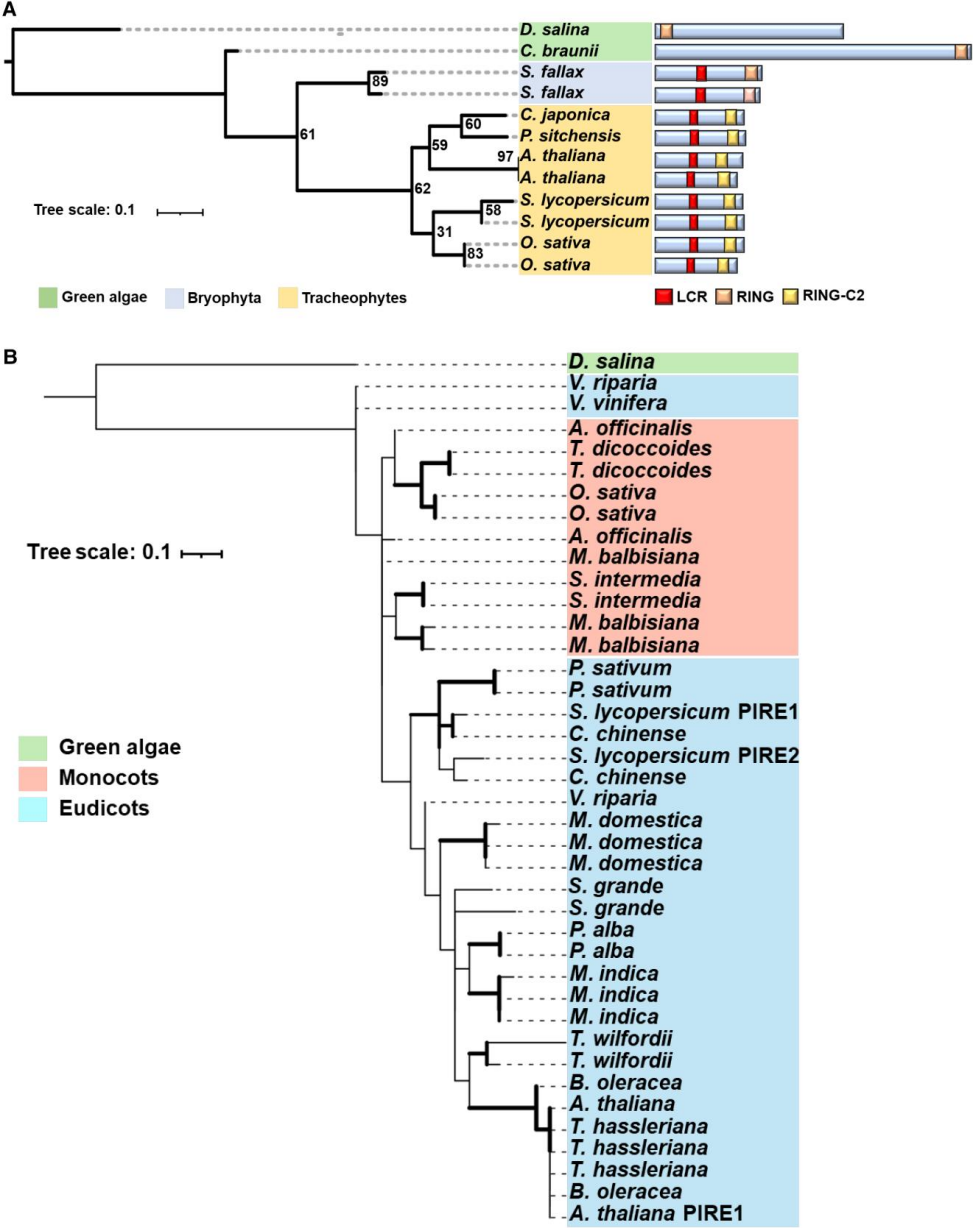
Homologs of the RING E3 ligase PIRE are present in the tracheophytes and expanded in angiosperms. **A)** PIRE homologs are detected in the Tracheophytes. Phylogeny of the RING domain from *Arabidopsis* PIRE and closest homologs throughout the plant kingdom. The phylogenetic tree was generated using the maximum likelihood method with a bootstrap value of 1,000 using IQtree. Right: Domain architecture of PIRE homologs, which contain a C-terminal modified RING-C2 domain, and a low complexity region (LCR) enriched in serine and glutamic acid residues in the central region of the protein. Scale bar represents branch length. **B)** Phylogeny of the RING domain from 39 PIRE protein homologs identified in 20 different plant species. The phylogenetic tree was generated using the maximum likelihood method with a bootstrap value of 1,000. Sequence alignments were generated utilizing Clustal Omega. Branches supported with bootstrap values above 70 have increased thickness. Scale bar represents branch length.

Next, we investigated the emergence of AtPIRE homologs across different algal and plant lineages. We required AtPIRE homologs to possess both the low complexity region and the C-terminally localized modified RING-C2 domain (Supplementary Fig. S1). Using a combination of BLASTP based on the RING-C2 domain coupled with the presence of the low complexity region, we identified 170 different PIRE homologs across 64 plant species (Supplementary Data Set 1). We identified 1 to 9 homologs per species. The 2 gymnosperms analyzed had only one *PIRE* homolog, 1 to 9 homologs were identified in angiosperms, and polyploids had the most homologs. These homologs contained a highly conserved modified RING-C2 domain region, which is important for zinc binding (Stone et al. 2005) (Supplementary Fig. S1; Fig. 1B). RING proteins identified in *Dunaliella salina,* in the phylum Chlorophyta (green algae) have N-terminal localized RING domains, but this domain does not contain the modified RING-C2 zinc-binding regions (Supplementary Fig. S1; Fig. 1A). Interestingly, *Chara braunii*, a member of the phylum Charophyta that emerged later than Chlorophyta, displayed a C-terminal localized ring domain; however, this domain does not have all the modified RING-C2 residues (Supplementary Fig. S1; Fig. 1A). PIRE-like architecture was also identified in Bryophyta, but members also lacked full RING-C2 residues (Supplementary Fig. S1). It was not until gymnosperms that both the PIRE architecture and complete modified RING-C2 residues appeared (Supplementary Fig. S1). PIRE homologs are expanded in angiosperms, and we were able to identify members in all analyzed monocot and dicot genomes (Fig. 1; Supplementary Data Set 1). Our analysis revealed that the complete PIRE protein architecture likely arose in gymnosperms.

### A conserved C-terminal RBOH residue regulates ROS production and abundance

**Figure 2.**
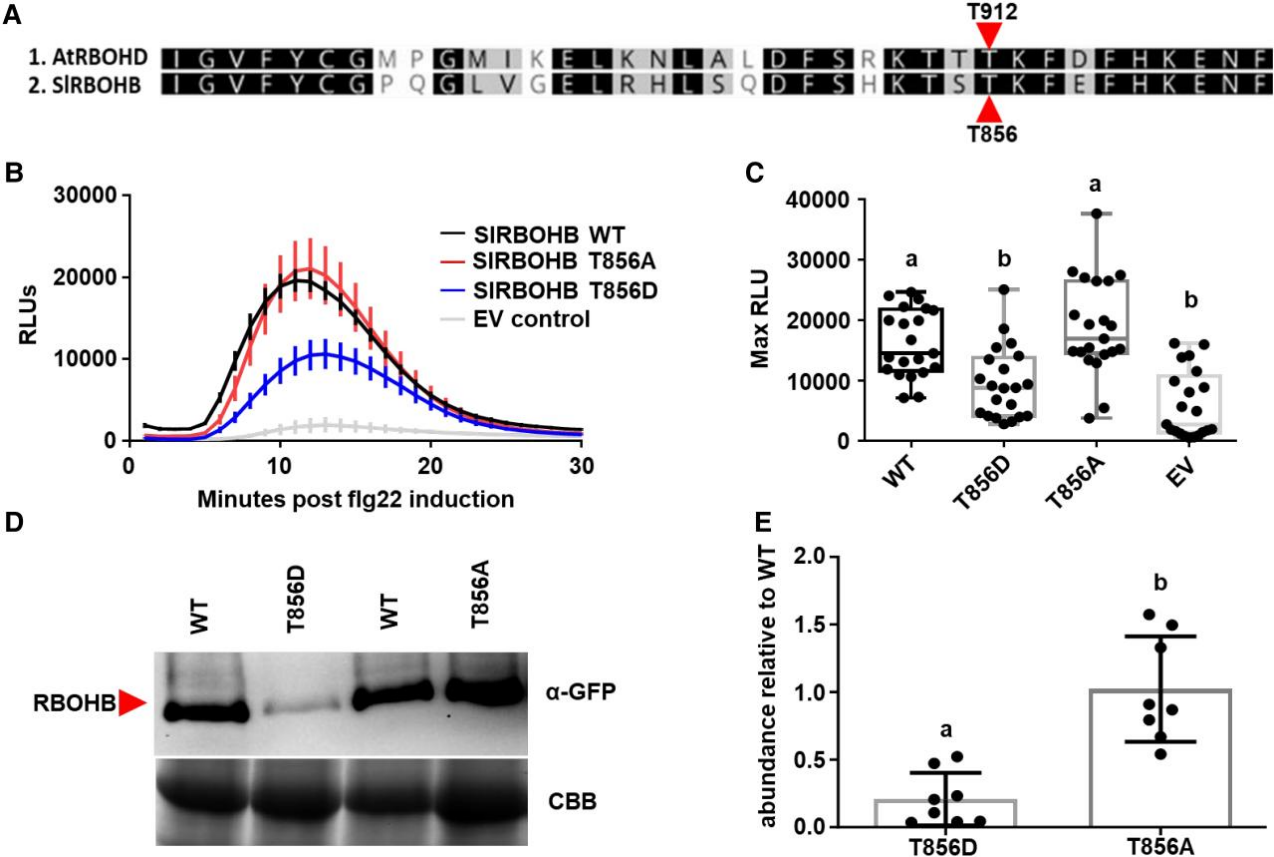
Mutations in conserved C-terminal residues *S. lycopersicum* RBOHB lead to changes in ROS production and protein accumulation. **A)** C-terminal amino acid alignment of the NADPH oxidases from Arabidopsis (AtRBOHD) and *S. lycopersicum* (SlRBOHB). The previously identified phosphorylated threonine 912 (T912) in AtRBOHD corresponds to threonine 856 (T856) in SlRBOHB. **B)** Different RBOHB variants were transiently expressed in *N. benthamiana*. Leaf disks were collected from *N. benthamiana* and treated with 100 nm flg22 to induce ROS production over 30 min. Results display the mean ± Se, *n* = 7 leaf disks. Phosphomimetic SlRBOHB^T856D^ has decreased the production of ROS compared with SlRBOHB^WT^ and SlRBOHB^T856A^. The assay was repeated 3 independent times. Graph represents 1 representative experiment. **C)** SlRBOHB^T856D^ ROS production is significantly lower than SlRBOHB^WT^ and SlRBOHB^T856A^ postflg22 induction as described above. Results display maximum relative light units (max RLU) of 3 independent experiments (*n* = 21 plants). The center line represents the median, the box highlights the upper and lower quartiles, and whiskers show minimum and maximum values. Statistical differences were determined by ANOVA with post hoc Tukey test (*P* < 0.0001). **D)** SlRBOHB protein abundance was visualized by anti-GFP immunoblot 48 h posttransient expression in *N. benthamiana*. SlRBOHB^T856D^ displayed reduced accumulation in *N. benthamiana* compared with SlRBOHB^WT^ and SlRBOHB^T856A^. The experiment was repeated 8 independent times. Picture represents 1 representative experiment. **E)** SlRBOHB protein accumulation was quantified from anti-GFP immunoblots utilizing Image Lab. Protein levels were first normalized using the Rubisco band from the Coomassie Brilliant Blue (CBB) gel, and then the relative intensity of each protein was compared with SlRBOHB^WT^ set to 1. The experiment was repeated 8 independent times (*n* = 8), error bars represent Sd. Statistical differences were calculated by Kruskal–Wallis test with Dunn's multiple comparison test (*P* = 0.0003). SlRBOHB^T856D^ has significantly lower protein accumulation than SlRBOHB^WT^ and SlRBOHB^T856A^.

Given the conservation of PIRE and the phosphorylated C-terminal residue corresponding to T912 in AtRBOHD (Lee et al. 2020), we sought to determine if additional plant NADPH oxidases are similarly impacted by the presence of this conserved residue. To this end, we investigated the *S. lycopersicum* SlRBOHB, which is the closest *S. lycopersicum* ortholog to AtRBOHD and has previously been linked with ROS production upon flg22 perception (Li et al. 2015). AtRBOHD residue T912 corresponds to SlRBOHB T856 (Fig. 2A). To validate the importance of T856 in regulating ROS production and stability of SlRBOHB, we generated both phosphonull (SlRBOHB^T856A^) and phosphomimic (SlRBOHB^T856D^) mutants of SlRBOHB fused to an N-terminal yellow fluorescent protein (YFP) epitope tag. Previous studies have shown that N-terminal tagging of RBOHD does not impact its function (Kadota et al. 2014; Lee et al. 2020). We transiently expressed these phosphomutants in *Nicotiana benthamiana* and induced PTI through flg22 treatment to measure production of ROS. Flg22-induced ROS was detected during the empty vector (EV) treatment due to endogenous RBOHB in *N. benthamiana* (Supplementary Fig. S2). ROS produced upon treatment with flg22 after transient expression of SlRBOHB was 10-fold higher than the EV control, demonstrating that we can use this assay to detect alterations in ROS after expression of additional RBOHs (Supplementary Fig. S2).

Transient expression of wild-type SlRBOHB (SlRBOHB^WT^) and SlRBOHB^T856A^ led to similar levels of ROS production postflg22 induction. However, transient expression of SlRBOHB^T856D^ led to a significant decrease in ROS production post flg22 induction (Fig. 2, B and C; the results from each statistical test are provided in Supplementary Data Set 2). Although there was a decrease in ROS production for SlRBOHB^T856D^, we did not detect changes in the temporal dynamics of ROS production with all transiently expressed SlRBOHB variants (Fig. 2B).

In Arabidopsis, phosphorylation of T912 leads to vacuolar degradation of AtRBOHD (Lee et al. 2020). Therefore, we hypothesized that decreased ROS production was due to reduced accumulation of the phosphomimic SlRBOHB^T856D^. We quantified the accumulation SlRBOHB and respective phosphomutants by western blot after transient expression in *N. benthamiana*. The accumulation of YFP-tagged SlRBOHB^WT^ and SlRBOHB^T856A^ was not significantly different from one another (Fig. 2, D and E). However, SlRBOHB^T856D^ displayed decreased accumulation by immunoblot analyses when compared with both SlRBOHB^WT^ and SlRBOHB^T856A^ (Fig. 2, D and E). These results are consistent with the regulation of AtRBOHD in Arabidopsis, where phosphorylation of T912 led to enhanced degradation of AtRBOHD (Lee et al. 2020). In our case, phosphomimic mutations of the corresponding residue in SlRBOHB, T856, also led to decreased accumulation of SlRBOHB during transient expression and in turn reduced production of ROS. These findings further support that phosphorylation of conserved residues plays an essential role in regulating NADPH oxidase abundance and ROS production.

### Accumulation of the SlRBOHB T856 phosphomimic is dependent on *PIRE* homologs

**Figure 3.**
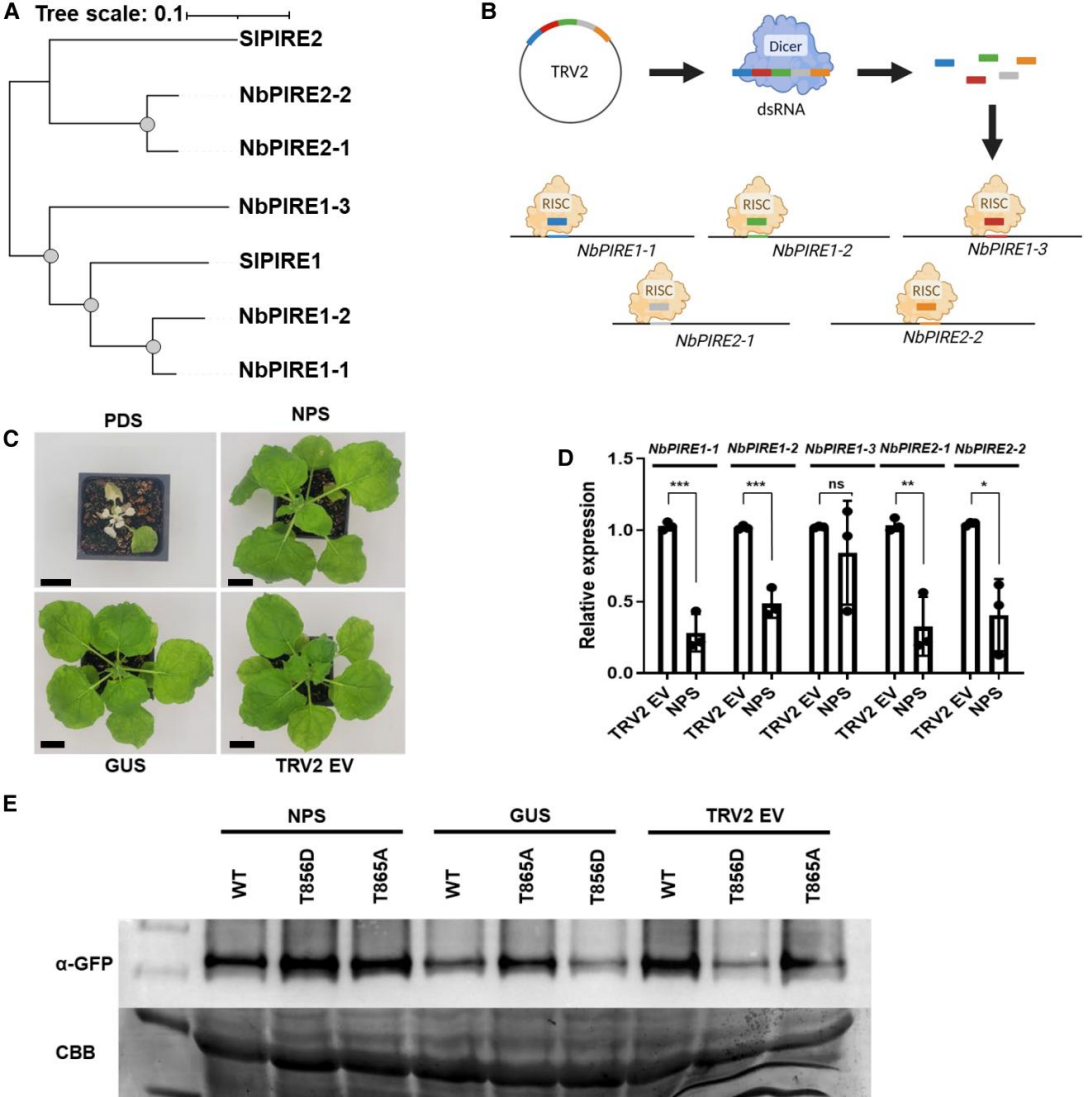
Changes in abundance of phosphomimetic SlRBOHB is dependent on *PIRE* homologs. **A)** Phylogenetic tree of *N. benthamiana* and *S. lycopersicum* PIRE homologs. Sequence alignments were generated utilizing the clustal omega program, and the mid-rooted phylogenetic tree was generated using maximum likelihood method with a bootstrap value of 1,000. Gray dots signify bootstraps higher than 80, Sl = *S. lycopersicum*, Nb = *N. benthamiana*. Scale bar represents branch length. **B)** Diagram of the stacked VIGS approach. Small (∼150 bp) regions of *NbPIRE* homologs were cloned into the TRV2 silencing vector, and then Agrobacterium carrying TRV1 and TRV2 were coinfiltrated into 2-wk-old *N. benthamiana*. The silencing fragments are converted into a long double-stranded RNA (dsRNA), which then get processed by dicer to generate short interfering RNAs (siRNAs) leading to depletion of 4 of 5 *NbPIRE* homologs (NPS construct). Diagram made in Biorender (agreement number JH281NIAW8). **C)** Images of *N. benthamiana* 2 wk post TRV inoculation via *A. tumefaciens*. The plant silenced for *PDS* displayed photobleaching and dwarfism. Black bars represent 3 cm. **D)**  *N. benthamiana*-silenced plants and controls were subjected to RT-qPCR to analyze PIRE expression levels. Relative expression was calculated compared with the Ef1α housekeeping gene. TRV2^NPS^ treated plants displayed significantly lower expression levels of *NbPIRE* homologs when compared with TRV2^EV^ control, except for *NbPIRE1-3*. Each data point represents the average of 1 biological replicate (*n* = 3 plants), and error bars represent Sd. Differences were detected by multiple *t*-tests (*P*-values: *Nbpire1-1* = 0.0007, *Nbpire1-2* = 0.0008, *Nbpire1-3* = 0.4369, *Nbpire2-1* = 0.0043, and *Nbpire2-2* = 0.0120). All experiments were performed 3 independent times. **E)** Wild-type SlRBOHB and phosphorylation mutants were transiently expressed in *N. benthamiana* 2 wk post TRV inoculation. Protein accumulation was visualized by anti-GFP immunoblotting. The image shows 1 representative experiment. Experiment was repeated 3 independent times. Silencing of *NbPIRE* homologs leads to enhanced accumulation for RBOHB^T856D^. TRV2^NPS^ plant displayed enhanced accumulation of RBOHB^T856D^ when compared with the TRV2^EV^ silencing control.

Next, we sought to determine if the abundance of SlRBOHB is dependent on *PIRE* homologs. There are 2 *S. lycopersicum PIRE* homologs, *SlPIRE1* and *SlPIRE2* (Fig. 1A). Using amino acid sequence alignments, we generated a phylogenetic tree to identify *N. benthamiana* homologs of *SlPIRE1* and *SlPIRE2*. Utilizing this method, we identified 5 homologs in *N. benthamiana*: 3 homologs of *SlPIRE1* (*NbPIRE 1-1, NbPIRE 1-2,* and *NbPIRE 1-3*) and 2 homologs of *SlPIRE2* (*NbPIRE 2-1* and *NbPIRE 2-2*) (Fig. 3A).

After identifying these *NbPIRE* homologs, virus-induced gene silencing (VIGS) was performed to ascertain their role in SlRBOHB abundance. We used tobacco rattle virus (TRV), which replicates via a double-stranded RNA intermediate, for VIGS in *N. benthamiana* (Senthil-Kumar and Mysore 2011; Bekele et al. 2019; Rössner et al. 2022). We simultaneously attempted to silence all *PIRE* homologs using a stacked VIGS approach, which incorporates small (150 bp) regions in a single TRV2 construct to silence *NbPire* homologs in parallel (TRV2^NPS^, *Nicotiana PIRE* Silencing [NPS]) (Fig. 3B) (Ahn et al. 2023).

We analyzed the abundance of SlRBOHB phosphomutants after silencing *NbPIRE*s. First, we infiltrated *N. benthamiana* plants with *Agrobacterium* carrying TRV2^NPS^, TRV2^Gus^, TRV2^EV^, and TRV2^PDS^. We used TRV2^PDS^ to silence phytoene desaturase (PDS), which interferes with the carotenoid biosynthesis pathway and induces photobleaching to visually assess silencing efficiency (Senthil-Kumar and Mysore 2011) (Fig. 3C). Two weeks post initial silencing with the TRV2 constructs, we performed a second set of infiltrations for *Agrobacterium*-mediated transient expression of SlRBOHB. To ensure *PIRE* silencing in *N. benthamiana,* we performed reverse transcription quantitative PCR (RT-qPCR) analysis. When compared with the TRV2^EV^ control, TRV2^NPS^ displayed significantly lower expression for all homologs except *NbPIRE1-3* (Fig. 3D). The lack of silencing of *NbPIRE1-3* could be due to the lower similarity to other *NbPIRE* homologs and reduced transcription of the last siRNA in the VIGS stack. Importantly, when multiple *PIRE* genes were silenced in *N. benthamiana*, the proteins SlRBOHB^WT^, SlRBOHB^T856A^, and SlRBOHB^T856D^ exhibited similar accumulation levels (Fig. 3E). In contrast, plants treated with TRV2^EV^ displayed lower levels of protein accumulation for SlRBOHB^T856D^ and similar protein accumulation for SlRBOHB^WT^ and SlRBOHB^T856A^ (Fig. 3E). These trends in protein accumulation were similar in TRV2^GUS^ treated plants. Taken together, the results with TRV2^NPS^ indicate changes in accumulation of the phosphomimetic SlRBOHB variants depend on *NbPIRE* in *N. benthamiana*.

### Gene editing of *SlPIRE1* leads to increased ROS production after flg22 treatment

**Figure 4.**
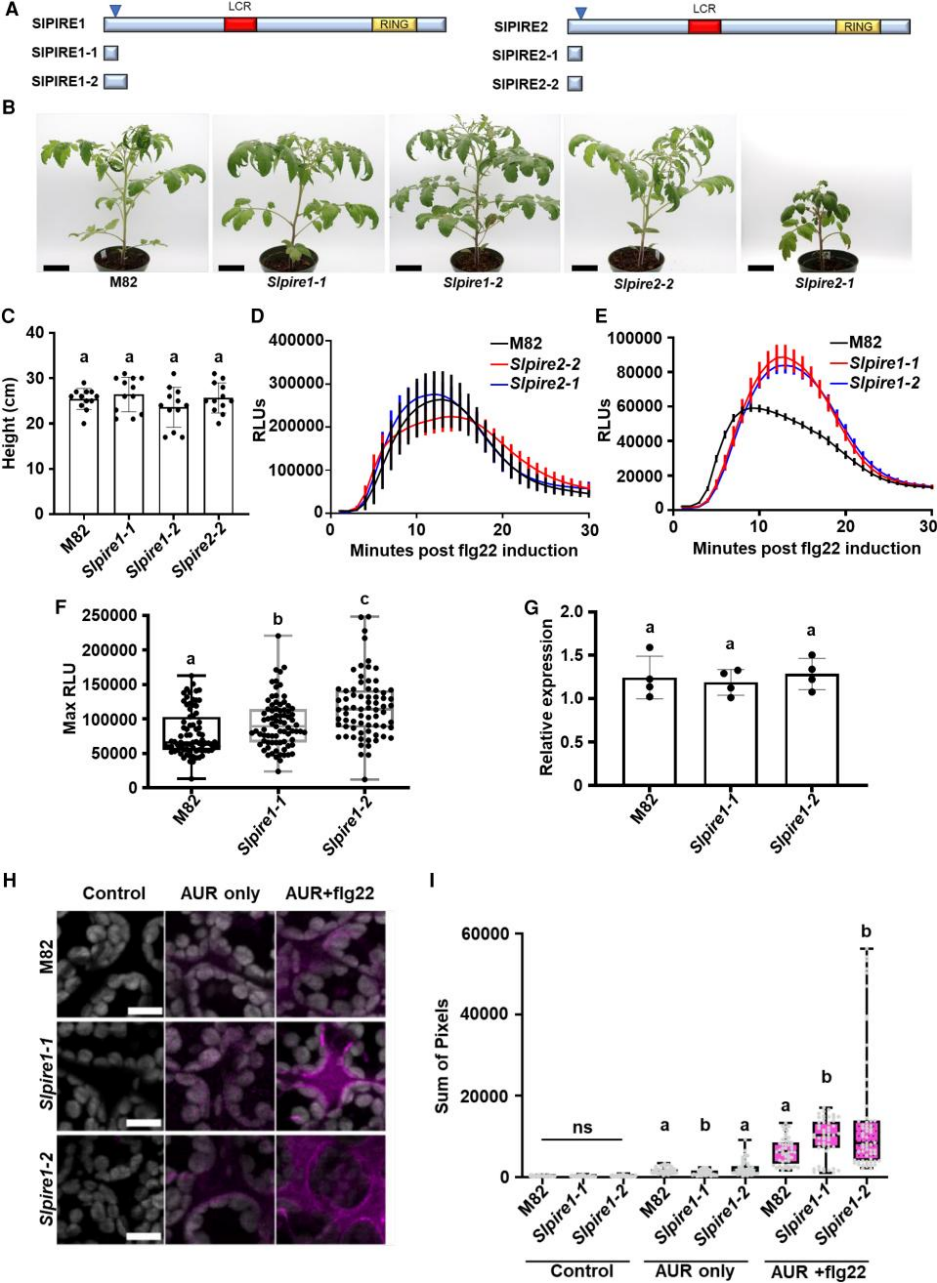
Editing tomato *SlPIRE1* results in enhanced production of ROS upon flagellin perception. **A)** Diagram of SlPIRE1 and SlPIRE2, arrows represent areas targeted by CRISPR/Cas9. Below the protein diagrams are the predicted truncated proteins generated from gene editing in *S. lycopersicum* cv M82. **B)** The *SlPIRE1* gene-edited lines did not display growth phenotypes in comparison with M82 (wild type [WT]) plants, under vegetative growth conditions. The *Slpire2* line 1 (*Slpire2-1*) displayed decreased growth compared with M82, but *Slpire2-2* displayed growth rates similar to M82. Black bars represent length of 6 cm. **C)** Height quantification of M82 and gene-edited lines. Heights were measured from soil to shoot apical meristem. *n* = 15 plants. Error bars represent Sd. Statistical analysis was performed by ANOVA with post hoc Tukey test (*P* = 0.2599). **D** and **E)** ROS production was analyzed in 4-wk-old M82, *Slpire1*, and *Slpire2* after treatment with 100 nm flg22. *Slpire2* gene-edited lines did not display changes in ROS production in comparison with M82 after flg22 treatment. *Slpire1* lines displayed enhanced ROS production post flg22 treatment compared with M82. *n* = 3 plants with 8 leaf disks per plant, error bars represent Sem. Graphs show 1 representative experiment. **F)** Quantification of ROS production in 4-wk-old M82, *Slpire1*, and *Slpire2* after treatment with 100 nm flg22. Results display maximum relative light units (max RLU). *Slpire1* lines produce significantly higher max RLU compared with M82 after flg22 treatment. *n* = 72 leaf disks over 3 sets of biological replicates (9 plants per genotype). Outliers were identified and removed using robust regression and outlier removal (ROUT) method (Q = 1%). The center line in the box represents the median, the box highlights the upper and lower quartiles, and whiskers show minimum and maximum values. Statistical differences were calculated by a 1-way ANOVA with post hoc Tukey test (*P* < 0.00010). **G)** Transcriptional expression of *Slrbohb* on tissue from M82, *Slpire1-1*, and *Slpire1-2*. RNA extraction and cDNA synthesis were performed on tissue collected from 4-wk-old tomato plants. Relative expression was calculated compared with the Ef1α housekeeping gene. There were no significant changes in transcription between M82, *Slpire1-1*, and *Slpire1-2* lines. Data point represents the average of 3 technical replicates per biological replicate (*n* = 4 plants), and error bars represent Sd. Kruskal–Wallis test with Dunn's multiple comparison test (*P* < 0.7697). **H** and **I)** ROS was visualized and quantified using the nonpermeable Amplex Ultra Red (AUR) stain 15 min postleaf infiltration with 100 nm flg22. AUR was visualized by confocal microscopy. Representative images of M82, *Slpire1-1*, and *Slpire1-2* with or without AUR and flg22 treatment. Image J was used to quantify the same size (1 × 1 cm) of 5 randomly selected regions per image. Three plants per genotype with 2 images per leaf were quantified, *n* = 6 images per genotype and treatment. Differences per treatment were calculated by Kruskal–Wallis test with Dunn's multiple comparison test (*P*-values: control = 0.3124, AUR only = 0.0005, and AUR flg22 = 0.0009). *Slpire1* lines exhibited significantly enhanced production of apoplastic ROS after induction with flg22. The center line in the box represents the median, the box highlights the upper and lower quartiles, and whiskers show minimum and maximum values. All experiments were repeated at least 3 independent times.

In Arabidopsis, *pire* knockouts exhibit enhanced ROS production and disease resistance (Lee et al. 2020). Therefore, we hypothesize that targeting *PIRE* homologs in other plants may confer enhanced ROS production. CRSIPR/Cas9 gene editing was used to generate *SlPIRE1* (Solyc03g113700) and *SlPIRE2* (Solyc06g071270) mutants. The CRISPR-P 2.0 web tool was used to select guide RNAs (gRNAs) specifically targeting the 5′ end of each homolog (Fig. 4A; Supplementary Fig. S3). Using these gRNAs, we generated 3 different constructs to transform *S. lycopersicum* cv. M82. Two constructs targeted the *SlPIRE1* and *SlPIRE2* genes independently, while the 3rd construct simultaneously targeted both genes. Two homozygous independent gene-edited lines for *SlPIRE1* (*Slpire1-1* and *Slpire1-2*) and *SlPIRE2* (*Slpire2-1* and *Slpire2-2*) (Fig. 4A) were obtained, which were verified by Sanger sequencing (Supplementary Fig. S4). These gene editing events led to the generation of frame-shift mutations leading to early stop codons. Cas9 was segregated out before conducting experiments with each line. After 4 rounds of transformation and 40 independent transformants, we were unsuccessful in generating a double mutant, indicating it may be lethal.

The gene-edited *Slpire1-1, Slpire1-2,* and *Slpire2-2* mutant lines displayed normal growth phenotypes in comparison with wild-type M82 plants (Fig. 4, B and C). However, *Slpire2-1* exhibited low germination, delayed germination, and smaller stature compared with wild-type M82 (Fig. 4, B and C; Supplementary Fig. S5). It is likely that smaller stature of *Slpire2-1* is due to delayed germination. We analyzed flg22-induced ROS production on wild-type and gene-edited lines. Both *Slpire2* lines and M82 produced similar levels of ROS after flg22 treatment (Fig. 4D). In contrast, *Slpire1-1* and *Slpire1-2* produced enhanced ROS compared with wild-type after flg22 treatment (Fig. 4, E and F). Therefore, we focused on *Slpire1* lines for future experiments. Both *Slpire1-1* and *Slpire1-2* also exhibited higher ROS after elicitation with chitin compared with M82, with *Slpire1-2* exhibiting statistically significantly higher ROS production (Supplementary Fig. S6). To determine if editing *SlPIRE1* impacted transcript accumulation of *SlRBOHB,* qPCR was performed (Fig. 4G). There was no difference in transcript accumulation of *SlRBOHB* between M82 and *Slpire1-1* or *Slpire1-2*, indicating ROS production is regulated posttranscriptionally by SlPIRE1 (Fig. 4G). We also utilized Amplex UltraRed reagent (AUR), a membrane impermeable reagent that directly interacts with H_2_O_2_ to quantify ROS levels (Ashtamker et al. 2007; Cohn et al. 2008). Both *Slpire1-1* and *Slpire1-2* displayed significantly enhanced apoplastic ROS accumulation after flg22 induction (Fig. 4, H and I). However, the baseline level of ROS in *Slpire1* edited lines and the dynamics of ROS production after flg22 treatment are not higher than wild-type M82. Taken together, our data show that *Slpire1* gene-edited lines specifically enhance apoplastic ROS production upon immune activation.

### *Slpire1* gene-edited lines exhibit increased resistance to foliar bacterial pathogens

**Figure 5.**
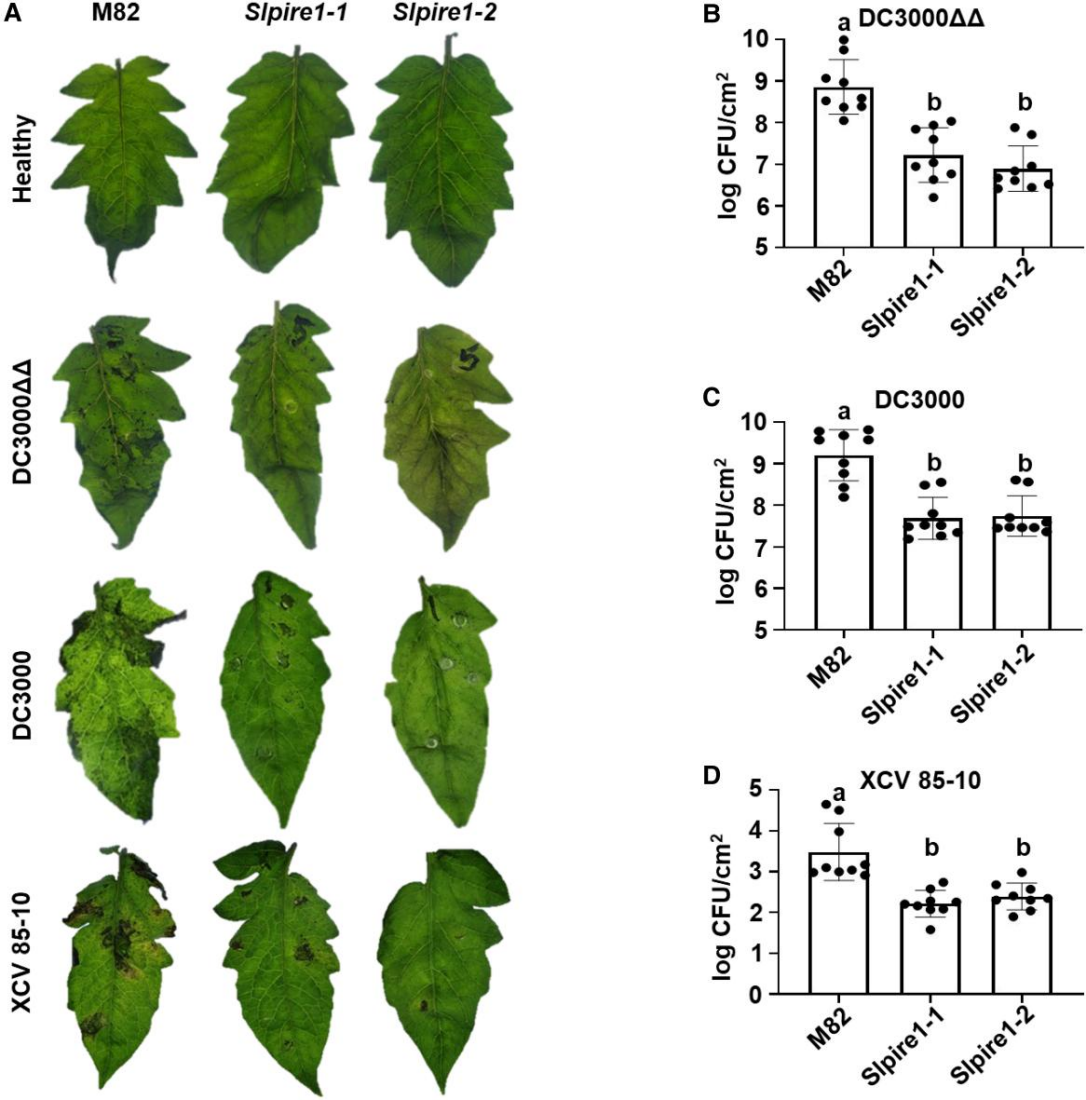
Editing *SlPIRE1* results in decreased disease symptoms and bacterial accumulation. **A)** Two independently gene-edited *SlPIRE1* lines (*Slpire1-1* and *Slpire1-2*) displayed reduced disease symptoms 3 dpi with *Pst* DC3000 Δ*avrPto*Δ*avrPtoB* (DC3000ΔΔ), 3 dpi for *Pst* DC3000 (DC3000), and 7 dpi for *X. campestris* pv. *vesicatoria* (XCV 85-10). Representative images of 9 plant infections. Images were digitally extracted for comparison. Black bars represent 1 cm length. Disease assays were performed 3 independent times per pathogen. Images show 1 representative experiment. To determine bacterial titers, leaf tissue was sampled 3 dpi for DC3000 and 7 dpi for XCV 85-10. Both *Slpire1-1* and *Slpire1-2* lines displayed decreased accumulation of DC3000ΔΔ **B)**, DC3000 **C)**, and XCV 85-10 **D)** compared with wild-type M82. *n* = 9 plants. Error bars represent Sd. Statistical analysis was performed by 1-way ANOVA with post hoc Tukey test (DC3000ΔΔ *P* < 0.0001, DC3000 *P* < 0.0001, and XCV85-10 *P* = 0.0423).

Since our gene-edited *Slpire1* lines displayed enhanced production of ROS upon immune activation, we sought to test their ability to resist pathogen infection. We performed syringe infiltration of 5-wk-old plants with the bacterial strain *P. syringae* pv. tomato DC3000 Δ*avrPto*Δ*avrPtoB* (DC3000ΔΔ), which contains mutations in 2 effectors and is less virulent than DC3000 (Lin and Martin 2005). M82 lacks resistance genes that recognize *avrPto* and *avrPtoB.* Both *Slpire1-1* and *Slpire1-2* exhibited reduced disease symptoms compared with wild-type M82 3 d postinfection (Fig. 5A). Furthermore, *Slpire1* lines displayed an 18-fold reduction in bacterial titers compared with the M82 control (Fig. 5B). Since DC3000ΔΔ exhibits attenuated virulence, we also utilized wild-type *P. syringae* DC3000 to challenge *Slpire1* lines in the M82 background. Similar to the infections for DC3000ΔΔ, infections with DC3000 led to decreased disease symptoms and 15-fold decrease in bacterial accumulation (Fig. 5, A and C). Next, we investigated the role of *SlPIRE1* in disease resistance to the causal agent of bacterial spot of tomato, *Xanthomonas campestris* pv. *vesicatoria* (XCV 85-10). Infections with *X. campestris* led to decreased disease symptoms, including reduced chlorosis in *Slpire1* lines compared with M82 at 7 d postinfection (Fig. 5A). Bacterial titers for *X. campestris* were 12-fold lower for both *Slpire1* lines when compared with the wild-type M82 control (Fig. 5D). Collectively, these data demonstrate that *SlPIRE1* mutants exhibit higher defense-induced ROS and increased disease resistance to foliar bacterial pathogens.

### *Slpire1* gene-edited lines do not impact disease caused by root-colonizing pathogens

To more comprehensively understand the role of *SlPIRE1* in plant defense, we also investigated its impact on root-invading pathogens. First, we challenged our gene-edited lines with *Ralstonia pseudosolanacearum* GMI1000, a soil-borne Gram-negative bacteria that causes bacterial wilt by colonization of xylem vessels (Salanoubat et al. 2002; Lowe-Power et al. 2016; Ingel et al. 2022). After petiole inoculation, plants were monitored for 14 d, and the disease index was measured. There were no significant differences in the disease index between wild-type M82 and *Slpire1-1* or *Slpire1-2* (Supplementary Fig. S7A). We also did not detect a difference after soil drench with *R. solanacearum* (Supplementary Fig. S7B). Next, we challenged *Slpire1-1* against the root-knot nematode *Meloidogyne javanica. M. javanica* invades the root tip and travels to the vascular cylinder to establish feeding sites comprised of giant cells. Here, the root-knot nematode will remain sedentary and complete its life cycle (Bartlem et al. 2014). We assessed nematode infection 7 wk postinoculation by extracting and quantifying *M. javanica* eggs from infected roots as a proxy for disease progress. We did not detect differences in egg accumulation between wild-type M82 and *Slpire1-1* (Supplementary Fig. S7C). Taken together, these data indicate that targeting *SlPIRE1* enhances foliar disease resistance without affecting root-colonizing pathogens.

## Discussion

For decades, *A. thaliana* has been utilized as an effective model system to study plant immunity. Arabidopsis is favored for its short life cycle and the extensive tools available for genetic manipulation (Rédei 1975; Nishimura and Dangl 2010). The discovery and investigation of Arabidopsis NLR and PRR immune receptors have provided insight into how plants recognize pathogens and activate immunity (Bent et al. 1994; Zipfel et al. 2006; Jones et al. 2024). Our knowledge of downstream immune signaling components stems from work in conducted Arabidopsis (Couto and Zipfel 2016; Yuan et al. 2023; Jones et al. 2024). These findings have laid the foundation for potential translation to crop plants (Li et al. 2024). For example, the Arabidopsis PRR elongation factor Tu receptor has been successfully introduced to multiple crop species, including tomato, rice, and sweet orange resulting in resistance to a variety of bacterial pathogens (Lu et al. 2015; Kunwar et al. 2018; Mitre et al. 2021). FLS2^XL^, a homolog of Arabidopsis FLS2, from wild grape can recognize *Agrobacterium,* a pathogen with divergent flg22 epitopes (Fürst et al. 2020). In this study, we examined the importance of the E3 ligase *PIRE*, which was originally identified in Arabidopsis, for its function in *S*. *lycopersicum.* By targeting *PIRE* homologs, we modulated the abundance of microbe associated molecular pattern (MAMP)-induced ROS in solanaceous plants and increased disease resistance to both *P. syringae* and *X. campestris*. *Slpire1* edited lines exhibited higher chitin-induced ROS and may also exhibit increased resistance to fungal pathogens. This study highlights another immune regulator originally identified in Arabidopsis with promise to enhance disease resistance in a variety of plant species.

The versatility of CRISPR/Cas9 to target genes across multiple plant systems has been leveraged to target susceptibility (S) genes for disease control (van Schie and Takken 2014; Bisht et al. 2019). Different classes of S genes include those involved in pathogen penetration, negative regulation of immune responses, and pathogen proliferation/dissemination (van Schie and Takken 2014). *PIRE* is a negative regulator of immune responses and regulates RBOH stability and ROS production in Arabidopsis (Lee et al. 2020). Our results indicate *SlPIRE1* is a promising S gene that also negatively regulates immune responses and foliar pathogen accumulation in tomato. There have been multiple examples of gene editing of negative immune regulators leading to increased disease resistance. Recently, gene editing of the xylem sap protein 10 (*XSP10*) and salicylic acid methyl transferase (*SISAMT*) led to tolerance to Fusarium wilt disease in tomato (Debbarma et al. 2023). Another well-known example is the negative immune regulator, Mildew Locus O (*MLO*). *MLO* mutants exhibit enhanced resistance to powdery mildew fungi in barley, wheat, and tomato (Jacott et al. 2021). However, production of higher order *mlo* mutants results in negative growth/yield penalties, including premature leaf senescence (Acevedo-Garcia et al. 2017; Jacott et al. 2021). Recently, the pleiotropic effects of *mlo* in *Triticum aestivum* have been circumvented by generating targeted mutations that lead to enhanced transcription *TaTMTB3,* a gene located directly upstream of *Mlo* on the chromosome, which uncouples negative growth phenotypes and resistance (Li et al. 2022). The most ideal S genes are those like *SlPIRE1,* where resistance is uncoupled from other pleiotropic effects. However, further characterization is necessary to ensure *SlPIRE1* lines do not display altered growth or yield phenotypes under field conditions.

*Slpire1* gene-edited lines did not display higher baseline apoplastic ROS or higher *SlROBHB* transcription but generated enhanced ROS production upon PRR activation. It is likely that higher baseline levels of SlRBOHB protein result in increased ROS production upon pathogen perception. In Arabidopsis, ROS production by RBOHD requires activation via calcium binding and phosphorylation (Li et al. 2014; Kadota et al. 2015; Zhang et al. 2018; Tian et al. 2019; Thor et al. 2020). Phosphorylation of Arabidopsis RBOHD at T912 leads to PIRE-mediated ubiquitination and vacuolar degradation, regulating the level of steady-state RBOHD (Lee et al. 2020). In our experiments, we observed significantly higher ROS production in *Slpire1* after induction with flg22 (Fig. 4, E to I). This is consistent with the requirement of RBOHs to be posttranslationally modified upon pathogen perception to generate ROS. Here, we show that mutations of the corresponding T912 residues in SlRBOHB (Fig. 2, D and E) or mutations in *SlPIRE1* (Fig. 3E) lead to changes in SlRBOHB accumulation. Taken together this suggests a model where SlRBOHB steady-state accumulation is enhanced by removal of *SlPIRE1*, which leads to increased ROS production upon pathogen perception. We attempted to analyze if SlRBOHB protein accumulation is impacted by a vacuolar degradation inhibitor, concanamycin A. However, we were unable to obtain reproducible results using transient expression in *Nicotiana.* Future experiments examining SlRBOHB localization during abiotic and biotic stress using tagged tomato lines will shed light onto prevalence of vacuolar degradation as a mechanism to regulate NADPH oxidase abundance.

E3 ligases are important as they provide specificity and bridge the interaction between the E2 ubiquitin ligase and their target protein (Sadanandom et al. 2012). Interestingly, neither of the *Slpire2* edited lines displayed alterations in defense-induced ROS production. This suggests that PIRE homologs in tomato do not have completely overlapping targets. Our inability to acquire the double mutant line for *Slpire1* and *Slpire2* suggests that removing both may be lethal. RBOHs play a role in plant development and response to stress (Kadota et al. 2015). In *Arabidopsis*, processes such as pollen tube growth, seed ripening, and formation of root hairs are dependent on *AtRBOHH, AtRBOHJ, AtRBOHB*, and *AtRBOHC,* respectively (Takeda et al. 2008; Müller et al. 2009; Kaya et al. 2014; Lassig et al. 2014). It is possible that *Slpire1* and *Slpire2* collectively regulate other RBOHs in *S. lycopersicum*. We observed delayed germination, reduced germination, and smaller height for the *Slpire*2-1 line. However, this was not observed in a second independent *Slpire2-2* line, indicating that *Slpire2-1* may have a secondary spontaneous mutation, CRISPR mediated mutation, or an epigenetic mark affecting germination.

Although *SlPIRE1* acts as an S gene toward the foliar pathogens *Pseudomonas* and *Xanthomonas*, it does not affect disease development for the root-colonizing bacteria *R. pseudosolanacearum* or root-knot nematode *M. javanica*. For pathogens with different life cycles, S genes can lead to enhanced susceptibility. For example, targeting the S gene *mlo* confers resistance to powdery mildew in wheat but enhances susceptibility to *Magnaporthe oryzae* pathotype *Triticum* (Gruner et al. 2020). However, there is evidence that activation of plant immune receptors can restrict both root-colonizing pathogens we tested. The NLR *Mi-1* has been used for decades to control resistance to root-knot nematodes within the *Meloidogyne incognita* group (MIG group) and is incorporated into many commercial tomato cultivars (Wubie and Temesgen 2019.). Transfer of the *EF-Tu* PRR to *S. lycopersicum* confers resistance to *R. pseudosolanacearum* in both greenhouse and field conditions (Lacombe et al. 2010; Kunwar et al. 2018). Targeting 2 enzymes involved in PRR-induced ROS, overexpression of the *RIPK* kinase or genome editing of the protein phosphatase *LOPP*, in the dwarf *S. lycopersicum* model plant, Micro-Tom, resulted in increased resistance to *R. pseudosolanacearum* (Wang et al. 2022). Roots are in contact with diverse microorganisms, and ROS can induce proliferation and induction of lateral roots, which are sites of entry for both *R. pseudosolanacearum* and *M. javanica* (Manzano et al. 2014; Tarkowski et al. 2023; Vailleau and Genin 2023; Hasan et al. 2024). It is possible that the inhibitory effect of increasing ROS in *Slpire1* is counteracted by alterations in root architecture. Alternatively, *SlPIRE1* may exhibit a different function or target in root vs. leaf tissue, consistent with the specificity achieved by RBOHs in different plant tissues (Chen and Yang 2020). ROS can also impact the function and assembly of the endogenous phyllosphere and rhizosphere microbiome (Pfeilmeier et al. 2021; Song et al. 2021). Thus, it will be important to analyze *Slpire1* edited lines under field conditions and for microbial assembly before deployment.

Pathogens frequently overcome single gene resistance, and no single R or S gene can serve as a silver bullet against all pathogens. A multilayered strategy that integrates resistance mechanisms at different stages of infection is a promising approach for durable disease resistance (Zhang and Coaker 2017). In *Oryza sativa*, expression of the PRR *Xa21* along with mutations of S genes including the transcription factor subunit *Xa5* and the sugar transporter *Xa13* leads to resistance against *X. oryzae* (Huang et al. 1997; Akter et al. 2024). Pyramiding a minimum of 2 adult plant resistance genes in *T. aestivum* resulted in adequate seedling stage resistance to stripe rust, caused by *Puccinia striiformis* f. sp. *tritici* (Wang et al. 2023). Targeting *PIRE,* in combination with other loci, could be a promising approach for effective pathogen control in the future.

## Materials and methods

### Plant growth conditions

*N. benthamiana* was obtained from the Staskawicz lab (Coaker et al. 2005). Tomato (*S. lycopersicum*) cv. M82 (LA3475) was obtained from the C.M. Rick Tomato Genetics Resource center at UC Davis. *N. benthamiana* was grown in Sunshine mix soil in a controlled environment chamber at 26 °C with a 16-h light/8-h dark photoperiod (180 *μ*mol m^−2^ s^−1^) set at 65% humidity. Plants were watered 3 times a week with fertilizer water (water mixed with Grow More 4-18-39, calcium nitrate, and magnesium sulfate) Four-week-old plants were used for *Agrobacterium*-mediated transient protein expression. Tomato plants (*S. lycopersicum* cv. M82) were grown in agronomy mix under controlled conditions at 26 °C and 12-h light/12-h dark photoperiod with 50% to 65% humidity. Plants were watered 3 times a week with fertilizer water. Five-week-old tomatoes were used for height measurements, ROS assays, and pathogen challenge. For height measurements, plants were measured from soil to the shoot apical meristem.

### Gene editing: guide design and construct generation

CRISPR gRNAs were designed using the CRISPR-P 2.0 web tool (http://crispr.hzau.edu.cn/CRISPR2). gRNAs were selected based on early targeting of the *SlPIRE1* and *SlPIRE2* genes, an on-target score higher than 0.4, and off-targets with scores primarily lower than 0.5 and in intergenic regions. gRNAs and off-target analysis are provided in Supplementary Tables S1 and S2. For single gRNA constructs, gRNAs were cloned into the pCR3-EF plasmid containing Cas9 (Fister et al. 2018) using Golden Gate assembly utilizing the *Bsa*I-HFv2 (NEB E1601S) restriction enzyme. For multiplex constructs targeting *SlPIRE1* and *SlPIRE2*, gRNA primers were used to amplify tRNA between both gRNAs (Xie et al. 2015). This gRNA–tRNA–gRNA multiplex was cloned into pCR3-EF plasmid using Golden Gate assembly as described above. pCR3-EF constructs containing the gRNAs were recombined into the pPZP200 destination vector (Hajdukiewicz et al. 1994). pPZP200 constructs containing gRNAs targeting *SlPire1, SlPire2*, and *SlPire1/SlPire2* were transformed into the cultivar M82 via *Agrobacterium* at the Innovative Genomics Institute (IGI Berkeley) and the transformation facility at University of Nebraska-Lincoln Center for Plant Sciences. Gene-edited lines were confirmed by Sanger sequencing for the targeted genes. Primers are listed in Supplementary Table S3.

### Sequence and phylogenetic analyses of PIRE homologs

Plant PIRE homologs were mined in NCBI utilizing BLASTP. We used mined homologs using the modified RING-C2 domain found in AtPIRE (AT3g48070). Utilizing this strategy, we identified 170 modified RING-C2 domain proteins with >70% amino acid (aa) similarly to the AtPIRE-modified RING domain in Charyophyta, Bryophyta, Gymnosperms, and Angiosperms. Full-length proteins were aligned utilizing Clustal Omega. Phylogenetic trees based on the RING domain of identified PIRE homologs were generated using the maximum likelihood method with a bootstrap value of 1,000 in IQ-TREE (Nguyen et al. 2015; Minh et al. 2020). Protein domains and low complexity regions were identified utilizing SMART (Letunic et al. 2021). For *N. benthamiana* PIRE homologs, we used the SlPIRE1 (Solyc03g113700) and SlPIRE2 (Solyc06g071270) aa sequences to mine for homologs. The NbPIRE1-1 (Niben101Scf04654g02005), NbPIRE1-2 (Niben101Scf07162g01018), NbPIRE1-(Niben101Scf02237g01001), and NbPIRE2-2 (Niben101Scf06720g01006) aa sequences were aligned using Clustal Omega. Phylogenetic trees were generated with the maximum likelihood method with a bootstrap value of 1,000 in IQ-TREE. Supplementary Data Set 1 includes the gene identifiers of all PIRE homologs. Supplementary Data Set 3 includes the alignment used for Fig. 1A. Supplementary Data Set 4 includes the newick phylogenetic tree for Fig. 1A. Supplementary Data Set 5 includes the alignment for Fig. 1B. Supplementary Data Set 6 includes the newick phylogenetic tree for Fig. 1B. Supplementary Data Set 7 includes the alignment used for Fig. 3A. Supplementary Data Set 8 includes the newick phylogenetic tree for Fig. 3A.

### Transient expression in *N. benthamiana*

For transient expression experiments, we generated constructs of SlRBOHB (Solyc03g117980) with C-terminal fusions to YFP. PCR-amplified cDNA was then directionally cloned into pENTR/D-TOPO (Invitrogen). Site-directed mutagenesis was performed on the pENTR/D-TOPO construct containing RBOHB to generate phosphomutants. pENTR/D-TOPO constructs were then sequenced before recombination into the pEarleyGate104 destination vector using Gateway technology (Earley et al. 2006). Constructs were electroporated into *Agrobacterium tumefaciens* (GV3101) (Weigel and Glazebrook 2006). Leaves of 4-wk-old *N. benthamiana* were infiltrated with the *Agrobacterium* containing each of the generated constructs (SlRBOHB^WT^, SlRBOHB^T856A^, SlRBOHB^T856D^, and EV) (OD_600_ = 0.6). Leaf tissue was harvested 48 h post infiltration (hpi) (3 leaf disks #3 cork borer [7 mm] per sample). Tissue was ground in 100 *µ*L of Laemmli buffer (Laemmli 1970). Protein samples were separated by SDS-PAGE, and immunoblotting was performed using anti-GFP-horse radish peroxidase (HRP) at a concentration of 1:5,000 (Miltenyi Biotec, 130-091-833, clone GG4-2C2.12.10). Image intensity quantifications were performed using Image Lab software (Image lab software version 6.1). All experiments were repeated at least 3 times with similar results. Data were analyzed by a Kruskal–Wallis test with a Dunn's test (*P* = 0.0003).

### Tomato transformation

#### Plant material and culture of explants

*S. lycopersicum* M82 seeds were surface sterilized in 20% (v/v) bleach solution containing 1 drop of Tween-20 for 15 min before rinsing 3 times with autoclaved water. The seeds were germinated in Petri dishes containing 30 mL of MS-based (Murashige and Skoog 1962) medium containing 30 g/L sucrose, 3.5 g/L phytagel (Sigma-Aldrich), and pH 5.8. Cultures were maintained at 26 °C under 16-h light/8-h dark photoperiod at 57–65 *µ*mol m^−2^ s^−1^ for 6 to 7 d.

#### *Agrobacterium*-mediated transformation of tomato

M82 cotyledon tissues excised from germinating seedlings were transformed through *Agrobacterium*-mediated transformation followed by selection and regeneration of transgenic lines using a modified protocol of Gupta and Van Eck (Gupta and Van Eck 2016). Transformation at the University of Nebraska, Lincoln, to generate *pire2* mutants was performed as previously described (Jones et al. 2004). Transformation at the IGI was performed as described below. Cotyledon explants were placed in the *Agrobacterium* (AGL1) suspension (OD_600_ of 0.4) on an incubating shaker (1.09 × g) at room temperature for 2 h. The tissues were then transferred to cocultivation medium and maintained at 21 °C for 4 d in the dark. The tissues were transferred to the first round of 2ZK selection medium containing 90 mg/L kanamycin. The tissues were maintained at 26 °C under 16 h light/8 h dark for 2 wk before transferring onto the 2nd round of 1ZK selection medium containing 90 mg/L kanamycin. Every 2 wk, the tissues were subcultured onto fresh medium. When shoots were ∼3 mm tall, they were excised from the cotyledon explants and transferred to selective RMK rooting medium containing 70 mg/L kanamycin. Plantlets were then transferred to soil once roots were established, and leaves touched the Phytatray lid.

### ROS burst assay

In *N. benthamiana,* leaf disks (4 mm diameter) were collected from plants transiently expressing SlRBOHB^WT^, SlRBOHB^T856A^, SlRBOHB^T856D^, and EV on the same leaf. Leaf disks were placed in water (200 *µ*L) for 20 h in CorningTM CorstarTM 96-well solid plates (Fisher #07-200-589) to recover before inducing with flg22. ROS was measured as previously described (Lee et al. 2020). The reaction solution contained 20 *μ*m L-012 (a luminol derivative from Wako Chemicals USA #120-04891), 10 mg mL^−1^ horseradish peroxidase (Sigma), and MAMPs including 100 nm flg22 (GeneScript, 95% purity) or 10 *μ*m hexaacetyl-chitohexaose (chitin) (Megazyme #O-CHI6). Light intensity was measured using a TriStar LB 941 plate reader (Berthold Technologies). In *S. lycopersicum*, 8 leaf disks (4 mm) were collected per plant per genotype (M82, *Slpire1-1*, *Slpire1-2, Slpire2-1,* and *Slpire2-2*). The assay was performed as described above. All experiments were repeated at least 3 times with similar results. Data from 3 experiments were combined. Whiskers show minimum and maximum values. Statistical differences were determined by ANOVA with post hoc Tukey test (*P* = 0.0001).

### VIGS of *NbPIRE* homologs

For VIGS, a gene block was generated (Twist Bioscience) containing 150-bp long regions of each *NbPIRE* homolog, cloned into pENTR/D-TOPO (Invitrogen), and recombined into TRV2 destination vector via LR clonase reaction. The TRV2 construct along with the TRV1 constructs were then electroporated into *Agrobacterium* (GV3101). Two-week-old *N. benthamiana* plants were coinfiltrated (OD600 = 0.4) with *Agrobacterium* containing TRV1 and TRV2 with one specific silencing region (TRV2^NPS^, TRV2^GUS^, TRV2^EV^, and TRV2^PDS^). *N. benthamiana* plants were allowed to grow for another 2 wk after infiltrations (4-wk-old plants) before transient expression. TRV2^PDS^ VIGS (Xu et al. 2019) plants served as a control to monitor silencing progress. Transient expression was performed as described above. Briefly *N. benthamiana* leaves were infiltrated with *Agrobacterium* containing the SlRBOHB variants described above. Leaf tissue was harvested 48 hpi and ground in 100 *µ*L of Laemmli buffer (Laemmli 1970). Protein samples were separated via SDS-PAGE gel, and immunoblotting was performed using anti-GFP-HRP at a concentration of 1:5,000 (Miltenyi Biotec, 130-091-833, clone GG4-2C2.12.10). Image intensity quantifications were performed using Image Lab software (Image lab software version 6.1). All experiments were repeated at least 3 times with similar results. Data were analyzed by ANOVA with post hoc Tukey test (alpha = 0.05).

### qPCR of VIGS-silenced plants

To examine the expression of *NbPire* homologs after silencing, we harvested 3-leaf punches with a #3 cork borer (7 mm) at the same time as we collected tissue for transient expression. Tissue was frozen and ground using liquid nitrogen. RNA was extracted from these plant samples with TRIzol (Fisher #15596018), following the manufacturer's instructions. DNase treatments for RNA preps were performed with RQ1 RNase-Free DNase (Promega #PR-M6101). cDNA synthesis was performed with the MMLV Reverse Transcriptase (Promega #PRM1705) kit. Primers for qPCR were designed using Primer3 (Untergasser et al. 2012) and are found in Supplementary Table S3. Gene expression was calculated using the Ct method and was normalized against the *N. benthamiana* EF1a housekeeping gene. qPCRs were performed with SsoFast EvaGreen Supermix with Low ROX (BioRad #1725211) in a 96-well white PCR plate (BioRad #HSP9601) according to the manufacturer's instructions. All experiments were repeated at least 3 times with similar results. Graphed data represent 3 biological replicates (plants), and differences were detected by 2-way ANOVA (alpha = 0.05).

### Visualization of apoplastic ROS by AUR

To visualize apoplastic ROS, 5-wk-old *S. lycopersicum* plant leaves were syringe-infiltrated with Amplex Ultra Red (AUR) or a combination with 100 nm flg22 (GeneScript, 95% purity). Leaf tissue was then visualized by confocal microscopy (Leica TCS SP8) 15 min postinfiltration. Imaging was performed with a 63× water immersion objective and 1 AU pinhole size. The image size was 1,024 × 1,024 pixels with a voxel size of 2.5 *μ*m and area size of 184.52 × 184.52 *μ*m. AUR was excited at 552 nm, with emission collected between 585 and 630 nm. The laser intensity was 3%. Chloroplast autofluorescence was gathered between 650 and 720 nm. Control images were taken from noninfiltrated tissue. Images were taken from 5 randomly selected regions of the same size (4 × 4 mm). Images were analyzed through ImageJ. Threshold: Default mode and minimum of 15 intensity were used for all images. Raw intensity density (RawintDen)—the sum of the values of the pixels in the image or selection—was used to quantify and compare. Three plants per genotype, 2 imaged per plant, and a total of 6 images for each treatment were quantified. Outliers were identified and removed using robust regression and outlier removal (ROUT) method (Q = 1%). Differences were calculated by a 1-way ANOVA with post hoc Tukey test.

### Statistical analyses

Each figure notes the statistical test performed. The results from each statistical test are provided in Supplementary Data Set 2.

### Disease assays

The *P. syringae* pv. *tomato* DC3000 (DC3000), *P. syringae* pv. *tomato* DC3000 *ΔavrPtoΔavrPtoB* (DC3000ΔΔ), and *X. campestris* pv. *vesicatoria* (XCV 85–10) were grown on NYG plates (Liu et al. 2013) with the appropriate antibiotics 2 d prior to infiltration. On the day of infection DC3000ΔΔ, DC3000, and XCV85-10 were resuspended on 5 mm MgCl_2_ (DC3000ΔΔ and DC3000: OD600 = 0.00005 and XCV85-10: OD600 = 0.0003). Five-week-old M82, *Slpire1-1*, and *Slpire1-2* plants were syringe inoculated with the pathogens listed above. We inoculated 3 to 4 leaves per plant per genotype (*n* = 5 plants per experiment). Leaf tissue was collected 3 d postinoculation (dpi). Images were collected from representative leaves. To measure the bacterial titers, we collected one #3 (7 mm) leaf disk per infected tissue. Leaf disks were ground up in 200 *μ*L of 5 mm MgCl_2_, and the solution was serially diluted from 10^−1^ to 10^−7^. Serial dilutions were plated on nutrient-glucose-yeast plates containing appropriate antibiotics along with 50 *μ*g/mL of cycloheximide. Colony counts were then performed after incubation at 28 °C for 48 h to determine the log CFU/cm^2^. All experiments were repeated at least 3 times with similar results. Statistical analysis was done by 1-way ANOVA with post hoc Tukey test (DC3000ΔΔ *P* = 0.0001, DC3000 *P* = 0.0001, and XCV85-10 *P* = 0.0423).

For infection with *R. pseudosolanacearum* GMI1000, we inoculated 21-d-old tomato plants with a cut-petiole approach by excising the lowest petiole and inoculating its surface with a 2-µL droplet of 5 × 10^5^ cfu/mL bacterial suspension (Khokhani et al. 2018). We rated disease progress for 14 d following disease index scale from 0 to 4, where 0 = 0 wilted leaves, 1 = 0.1% to 25% of wilted leaves, 2 = 25.1% to 50% of wilted leaves, 3 = 50.1% to 75% of leaflets wilted, and 4 = 75.1% to 100% of wilted leaves (Khokhani et al. 2018)

*M. javanica* infections were performed as previously described (Yimer et al. 2023). Briefly, sterile nematode eggs were collected from previously infected tomato plant cultures (Mormor Verte variety) using a 10% bleach solution. Eggs were allowed to hatch at 27 °C to collect J2 stage nematodes. Collected J2 stage nematodes where then washed on a 50-mL vacuum filtration unit (e.g. 22 *µ*m, Thermo Scientific Nalgen Filtration Product, Rochester, NY, USA) using sterile water. J2 numbers were obtained at this point before infecting 4-wk-old plants (M82 and Slpire1-1). Infected plants were harvested 7 wk postinfection. Eggs were collected from infected plants by using a 10% bleach wash and using sieves of mesh #200 (75 *µ*m) and mesh #500 (25 *µ*m) to separate the eggs. Egg counts were performed under a dissecting microscope (Yimer et al. 2023). All experiments were repeated twice with similar results, and data were analyzed for significant differences by *t*-test.

### Accession numbers

Sequence data from this article can be found in the GenBank/EMBL data libraries under accession numbers: AtPIRE (AT3g48070), SlPIRE1 (Solyc03g113700), SlPIRE2 (Solyc06g071270), NbPIRE1-1 (Niben101Scf04654g02005), NbPIRE1-2 (Niben101Scf07162g01018), NbPIRE1-3 (Niben101Scf07162g01018), NbPIRE2-1 (Niben101Scf02237g01001), and NbPIRE2-2 (Niben101Scf06720g01006).

## Supplementary Material

koaf049_Supplementary_Data

## Data Availability

All raw data and experimental repeats have been deposited in Zenodo https://doi.org/10.5281/zenodo.13119655.

## Supplementary data

The following materials are available in the online version of this article.

**Supplementary Data Set 1.** Identified PIRE homologs.

**Supplementary Data Set 2.** Statistical analysis.

**Supplementary Data Set 3.** Fasta alignment for Fig. 1A.

**Supplementary Data Set 4.** Newick phylogenetic tree for Fig. 1A.

**Supplementary Data Set 5.** Fasta alignment for Fig. 1B.

**Supplementary Data Set 6.** Newick phylogenetic tree for Fig. 1B.

**Supplementary Data Set 7.** Fasta alignment for Fig. 3A.

**Supplementary Data Set 8.** Newick phylogenetic tree for Fig. 3A.

**Supplementary Table S1.**  *SlPIRE1* gRNA analysis.

**Supplementary Table S2.**  *SlPIRE2* gRNA analysis.

**Supplementary Table S3.** PCR primers.

**Supplementary Figure S1.** Alignment of the modified RING-C2 domain found in green algae, bryophytes, gymnosperms, and angiosperms.

**Supplementary Figure S2.** Transient expression of SlRBOHB can be differentiated from the endogenous NbRBOHB burst after flg22 induction.

**Supplementary Figure S3.** Validation of *Slpire* gene-edited lines by DNA sequencing.

**Supplementary Figure S4.** Alignments of the amino acid translations for gene-edited *Slpire* lines.

**Supplementary Figure S5.** Germination percentages for M82, *Slpire1-1*, *Slpire1-2*, *Slpire2-1*, and *Slpire2-2*.

**Supplementary Figure S6.** ROS production after induction with chitin.

**Supplementary Figure S7.** Disease measurements for *R. pseudosolanacearum* GMI1000 strains and *M. javanica* strains on M82 (wild-type) and *Slpire1* edited lines.

## Dive Curated Terms

The following phenotypic, genotypic, and functional terms are of significance to the work described in this paper:

FLS2 Gramene: AT5G46330
FLS2 Araport: AT5G46330

PUB13 Gramene: AT3G46510
PUB13 Araport: AT3G46510

leaf AmiGo: PO:0025034

RBOHB Gramene: Solyc03g117980
RBOHB Araport: Solyc03g117980

NADPH CHEBI: CHEBI:16474

protein CHEBI: CHEBI:36080

XA21 Gramene: LOC_Os11g36180
XA21 Araport: LOC_Os11g36180
