# Supplementary material for: Gene editing of the E3 ligase PIRE1 fine-tunes reactive oxygen species production for enhanced bacterial disease resistance in tomato
Source: Plant Cell. 2025 May 30;37(5):koaf049. doi: 10.1093/plcell/koaf049 (PMC12124405; doi:10.1093/plcell/koaf049)
Supplement: koaf049_Supplementary_Data [file koaf049_supplementary_data.zip › Supplementary materials.pdf]

Supplementary materials:

Dataset S1, Tables S2-S3, Figures S1-S7

Supplementary Tables

Supplementary Table S1. *SlPIRE1* gRNA analysis

Table displays the position of the gRNA, gRNA sequence, and off-target sites with scores.

Slpire1 guide

guide1 on-score: 0.4078					
postion: SL3.0ch03:-65252750					
guide sequence: TTGGGGTTAGAGGAAGCAATAGG					
number of offtarget sites: 91					
top 20 genome-wide off-target sites					
Sequence	Off-score	MMs	Locus	Gene	Region
TTGGGATTAAAGGAAGCAATGGG	0.933	2MMs	SL3.0ch04:-39722100	gene:Solyc04g049130.3	intron
TTAGAGTTAGAGAAAACAATAGG	0.6	4MMs	SL3.0ch08:-48082971		Intergenic
TTGGGCTGAGAAGAAACAATAGG	0.467	4MMs	SL3.0ch02:-15727873	gene:Solyc02g014170.3	intron
TTGGGGCGAGAAAAAGCAATAGG	0.434	4MMs	SL3.0ch02:-32851223		Intergenic
TTGGGGAAAGAGGAAGAAATAGG	0.327	3MMs	SL3.0ch03:-30995822	gene:Solyc03g059316.1	intron
TTGGGGAAAAAGGAAGAAATGGG	0.305	4MMs	SL3.0ch09:-14872449		Intergenic
TTGGGGAAAAAGGAAGAAATGGG	0.305	4MMs	SL3.0ch04:-13773084		Intergenic
TTGGGGAAAAAGGAAGAAATGGG	0.305	4MMs	SL3.0ch09:-46235736		Intergenic
TTGGGGAAAAAGGAAGAAATGGG	0.305	4MMs	SL3.0ch10:+28569421		Intergenic
TTGGGGAAAAAGGAAGAAATGGG	0.305	4MMs	SL3.0ch08:+12339840		Intergenic
TTGGGGAAAAAGGAAGAAATGGG	0.305	4MMs	SL3.0ch09:+48321502		Intergenic
TTGGGGTGAAAGAAAGAAATGGG	0.295	4MMs	SL3.0ch01:+55850960		Intergenic
TTGAGGTTAAGGGAAGCATTGGG	0.294	4MMs	SL3.0ch08:-316739	gene:Solyc08g005430.3	utr
TTGGGGCTAGAACAAGCAATAGG	0.27	3MMs	SL3.0ch09:+66041209		Intergenic
TTGGAGGAAGAGGAGGCAATGGG	0.265	4MMs	SL3.0ch09:-7380213	gene:Solyc09g014980.3	CDS
TTGGGATTTGAACAAGCAATAGG	0.236	4MMs	SL3.0ch09:+41852222		Intergenic
TGGGGGTATGAGGAAGCAAATGG	0.227	4MMs	SL3.0ch03:-64463534	gene:Solyc03g112590.3	CDS
TTGGGGTTAGAAAAAGAAAAAGG	0.226	4MMs	SL3.0ch03:-38929962		Intergenic
TTTAGGGAAGAGGAAGCAATTGG	0.212	4MMs	SL3.0ch03:+10672548		Intergenic
TTGGGGGAAAAGGAAGAAATAGG	0.205	4MMs	SL3.0ch06:+5875458		Intergenic

Supplementary Table S2. ***SlPIRE2*** **gRNA analysis**

Table displays the position of the gRNA, gRNA sequence, and off-target sites with scores.

Slpire2 guide

guide1 on-score: 0.4317					
postion: SL3.0ch06:-43989211					
guide sequence: TGGAATTAGAGGAAGCGACAGGG					
number of offtarget sites: 29					
top 20 genome-wide off-target sites					
Sequence	Off-score	MMs	Locus	Gene	Region
TGGAACTATAAGAAGCAACAAGG	0.453	4MMs	SL3.0ch02:-54049947	gene:Solyc02g092290.3	CDS
TGGAATTATTGGAAGCAACATGG	0.439	3MMs	SL3.0ch02:+39993027	gene:Solyc02g069580.3<br>gene:Solyc02g069570.3	CDS
TGGAATTTGAAGAAGAAACAGGG	0.4	4MMs	SL3.0ch03:+2095648		Intergenic
TTGAATGAGAGGAAGAAACAGGG	0.349	4MMs	SL3.0ch11:-47658739		Intergenic
TTGAATGAGAGGAAGAAACAGGG	0.349	4MMs	SL3.0ch11:-47655215		Intergenic
TGGAAATTGAAGAAGCAACAGGG	0.347	4MMs	SL3.0ch07:-815344	gene:Solyc07g005970.3	intron
TGGAAGAAGAGGAAGAGACTAGG	0.3	4MMs	SL3.0ch03:+46056661		Intergenic
TGGAGATAGAGGAGGAGACAAGG	0.291	4MMs	SL3.0ch05:+57118290		Intergenic
TGGGATTTGAGGAAAAGACATGG	0.252	4MMs	SL3.0ch07:+54493200		Intergenic
TGGGGTTAGAGGAAGCAATAGGG	0.194	4MMs	SL3.0ch03:-65252755	gene:Solyc03g113700.3	CDS
AGGAAGTGGAGGAAGCGAAATGG	0.18	4MMs	SL3.0ch04:-20397120		Intergenic
TAGTATTAGAGGAAGCAACAAAG	0.13	3MMs	SL3.0ch07:-32490648		Intergenic
TGGAATTAGAAAAGGCGACAGAG	0.113	3MMs	SL3.0ch01:-31110938		Intergenic
TGCGCTTAGAGAAAGCGACATGG	0.112	4MMs	SL3.0ch09:+28927574	gene:Solyc09g031640.1	CDS
TGGAATTGGAGAGAGCCACATGG	0.105	4MMs	SL3.0ch12:+19685978	gene:Solyc12g077540.2	CDS
TGGAGTTCGAAGAAGTGACAAGG	0.095	4MMs	SL3.0ch11:+38553805		Intergenic
TGAAATTGGAGGAAGAGAGAGGG	0.069	4MMs	SL3.0ch06:-31118023	gene:Solyc06g048430.3	intron
TCGATATAGAGGAAGTGACAAGG	0.067	4MMs	SL3.0ch11:-31892066		Intergenic
TGGAAGTAGAAGAAGCAATAGAG	0.064	4MMs	SL3.0ch04:-22943006		Intergenic
TGGAATTAGGGGTAGTGACAAGG	0.051	3MMs	SL3.0ch02:+35078725	gene:Solyc02g062750.3	intron

Supplementary Table S3. Table of primers used for qPCR, genotyping, and cloning.

Primer	Sequence	Details
Ef1a forward	AGCTTTACCTCCCAAGTCATC	qPCR primer
Ef1a reverse	AGAACGCCTGTCAATCTTGG	qPCR primer
NbPire1-1 forward	AACGGTTCGATCCCTATTGTG	qPCR primer
NbPire1-1 reverse	GTTCCTCCTTCAAGCCTTTG	qPCR primer
NbPire1-2 forward	CAGCGGTTCGATCCCTATTG	qPCR primer
NbPire1-2 reverse	TTTGGTTCCTCCTTCAAGCC	qPCR primer
NbPire1-3 forward	CAATCCCTAACGCTTCCATCC	qPCR primer
NbPire1-3 reverse	GCTTGCACTGCTTCAACTTGG	qPCR primer
NbPire2-1 forward	ATCGCCAATGCTTCAATCCC	qPCR primer
NbPire2-1 reverse	CATTCAATTCCTCATTGGAGCC	qPCR primer
NbPire2-2 forward	CCATGCTTTCCTCTAACCCC	qPCR primer
NbPire2-2 reverse	TACCCCACCATTCAATTCCTC	qPCR primer
SlRBOHB forward	ATGATAGAGCGTCGTACAGTGG	qPCR primer
SlRBOHB forward	CCGATGTCAGTAGATTCCGGT	qPCR primer
Cas9 check F	AGTTCCTGGTCCACGTACATATCC	Genotyping
Cas9 check R	ACAGAGAGATGATCGAGGAACGG	Genotyping
PIRE1-GE check F	GTTGAATTGAGGGGCAATTACG	Genotyping
PIRE1-GE check R	GATCTTATGCTACCTTGTGAAAGC	Genotyping
PIRE2- GE check F	TTCCATAAACAATAGGGTTGCAG	Genotyping
PIRE2- GE check R	CTTGCTCTGCTTCAACTTAGCC	Genotyping
SlRBOHB Topo F	CACC ATGCAAAATTCGGAAAATC	Cloning Slrbohb
SlRBOHB Topo R	TCAAAAATTTTCTTTATGGAAATC	Cloning Slrbohb
SlRBOHB T856D F	AAGACATCCGACAAGTTTG	Site directed mutagenesis Slrbohb
SlRBOHB T856D R	CAAACTTGTCGGATGTCTT	Site directed mutagenesis Slrbohb
SlRBOHB T856A F	AAGACATCCGCCAAGTTTG	Site directed mutagenesis Slrbohb
SlRBOHB T856A R	CAAACTTGGCGGATGTCTT	Site directed mutagenesis Slrbohb

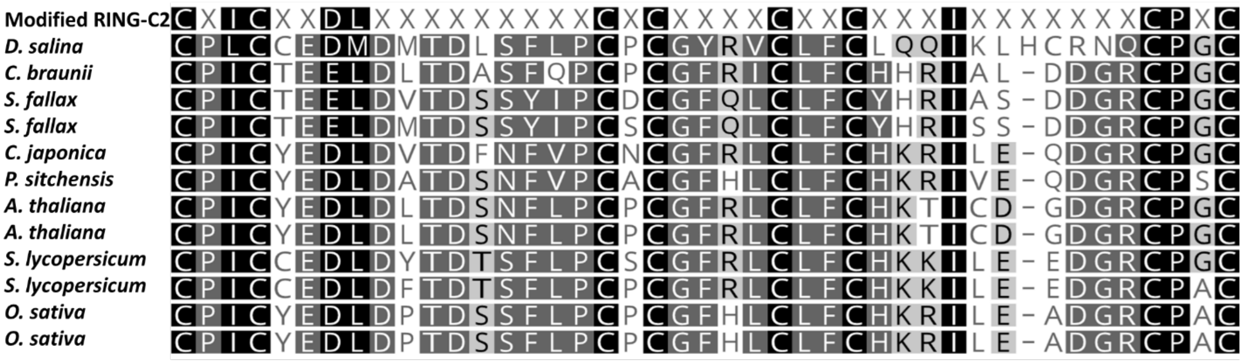

Supplementary Figure S1. Alignment of the modified RING-C2 domain found in Green Algae, Bryophytes, Gymnosperms, and Angiosperms.

(A) Alignment of a subset of identified modified RING-C2 ubiquitin ligases. The alignment was generated utilizing clustal omega. The top sequence (Modified RING-C2) represents the previously described amino acids found in RING-C2s in Arabidopsis which are important for the interaction with zinc.

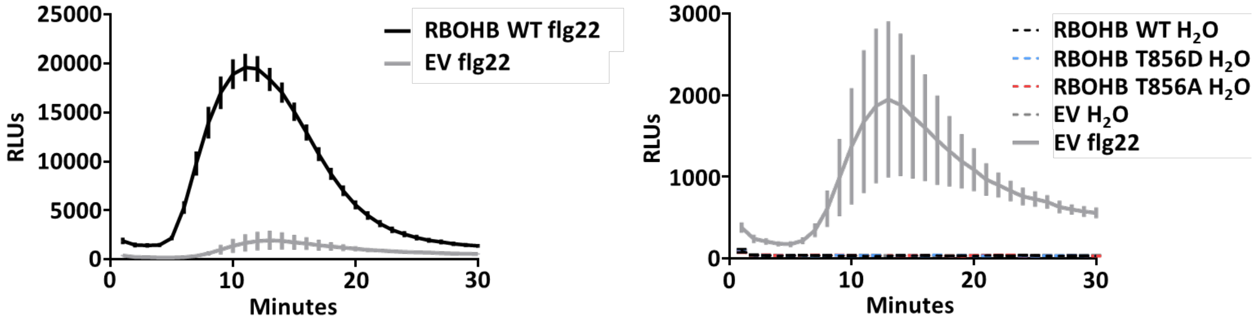

Supplementary Figure S2. Transient expression of *SlRBOHB* can be differentiated from the endogenous *NbRBOHB* burst after flg22 induction.

Left Panel: ROS burst is induced in *Nicotiana benthamiana* during flg22 treatment after infiltration with *Agrobacterium* carrying empty vector (EV, solid grey) and the burst is higher after transient expression of RBOHB from *S. lycopersicum* (solid black). Leaf disks were collected from *N. benthamiana* and treated with 100nM flg22 to induce ROS production over 30 minutes. Results display the mean ±SE, n=7 leaf disks, RLU = relative light units. Water controls display no ROS burst. Right Panel: ROS burst after flg22 treatment after infiltration with *Agrobacterium* carrying EV (solid grey). All water treatments did not induce a ROS burst (dotted lines). Note the difference in scale for RLUs between both panels. Experiments were repeated three times with similar results.

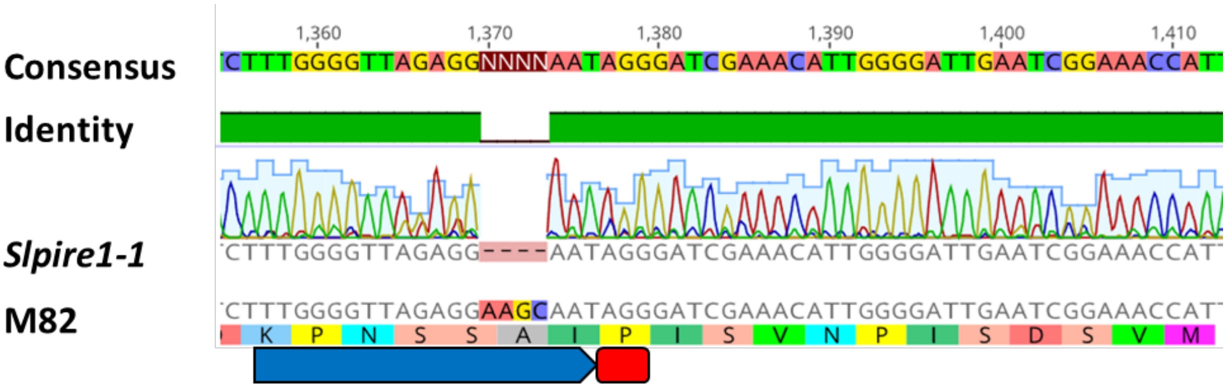
Consensus
Identity
Slpire1-1
M82

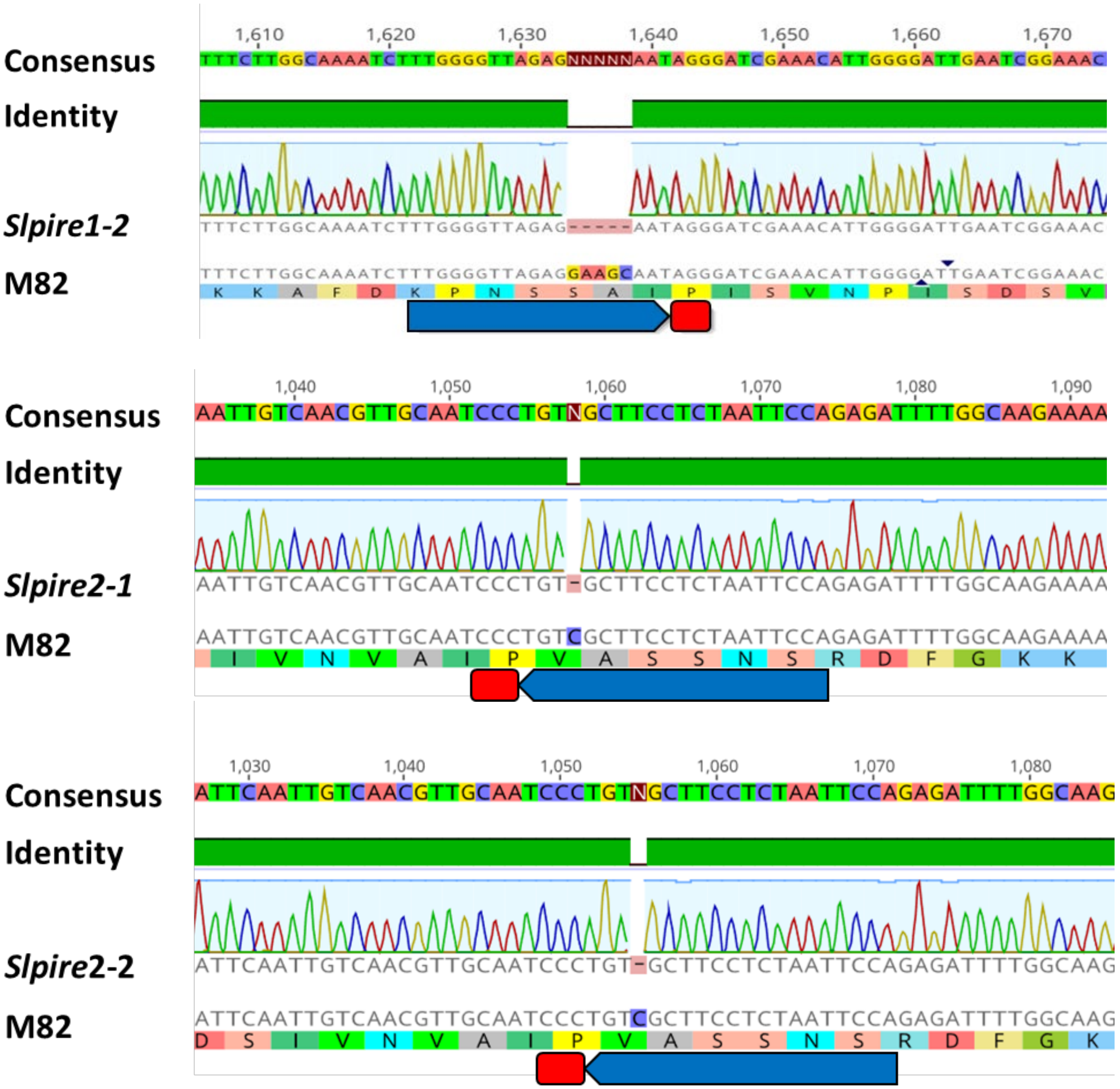
Consensus
Identity
Slpire1-2
M82
Consensus
Identity
Slpire2-1
M82
Consensus
Identity
Slpire2-2
M82

Supplemental Figure S3. Validation of *Slpire* gene edited lines by DNA sequencing. Sequencing results for *Slpire1* and *Slpire2* gene edited lines. Gene editing occurred in the *Solanum lycopersicum* cultivar ‘M82’. Blue arrow highlights the gRNA utilized while the red box represents the protospacer adjacent motif (PAM)**.**

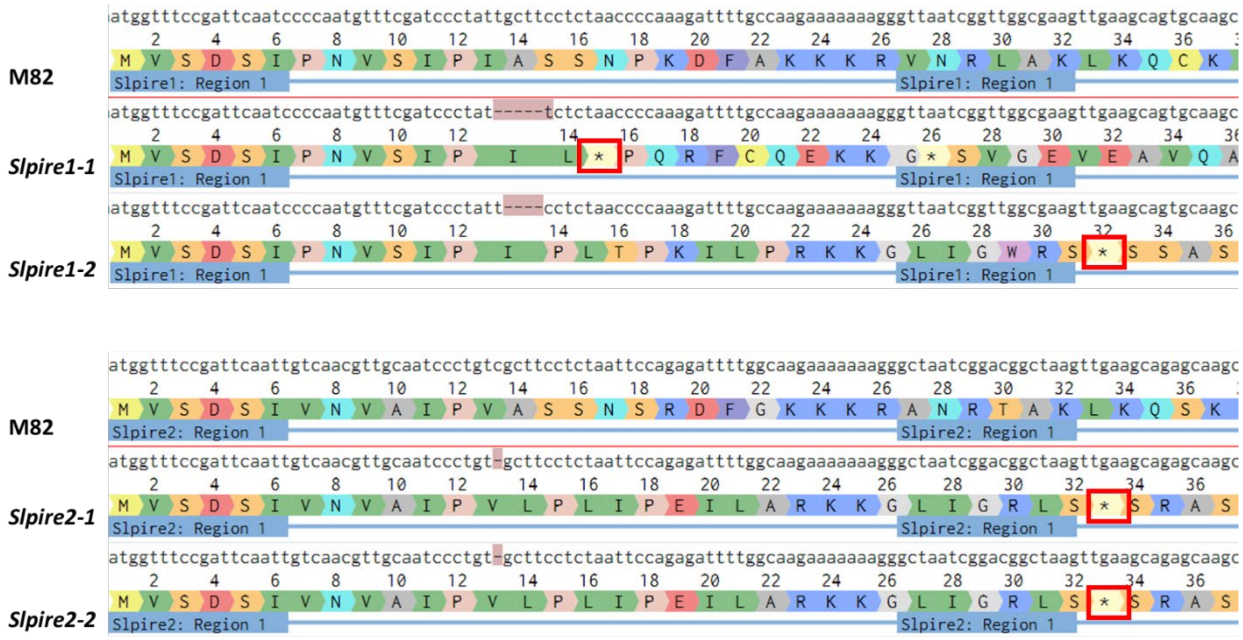

Supplementary Figure S4. Alignments of the amino acid translations for *Slpire* mutant lines.

Amino acid alignments displaying the frameshift mutations generating early stop codons on gene edited lines (Stop codons are highlighted by red boxes). Alignment was generated using Clustal omega. Top: *Slpire1* edited lines, bottom: *Slpire2* edited lines.

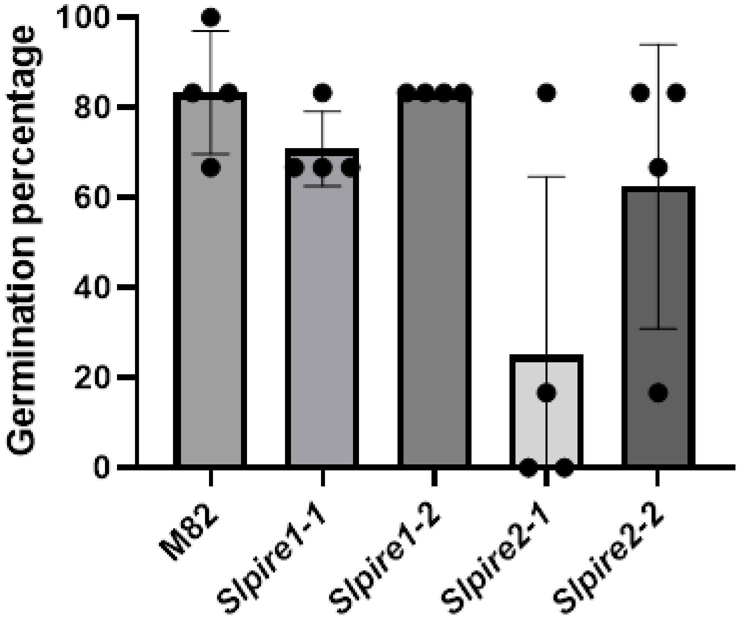

Supplementary Figure S5. Germination percentage for M82, *Slpire1-1*, *Slpire1-2, Slpire2-1,* and *Slpire2-2*.

Germination percentage for M82 and *Slpire* gene edited lines. Germination percentages were measured four independent times. The percentages represent the number of seeds that germinated from a total number of eight seeds planted (N=36 seed, eight seeds per replicate, four independent experiments). *Slpire2-1* line displayed reduced germination rates.

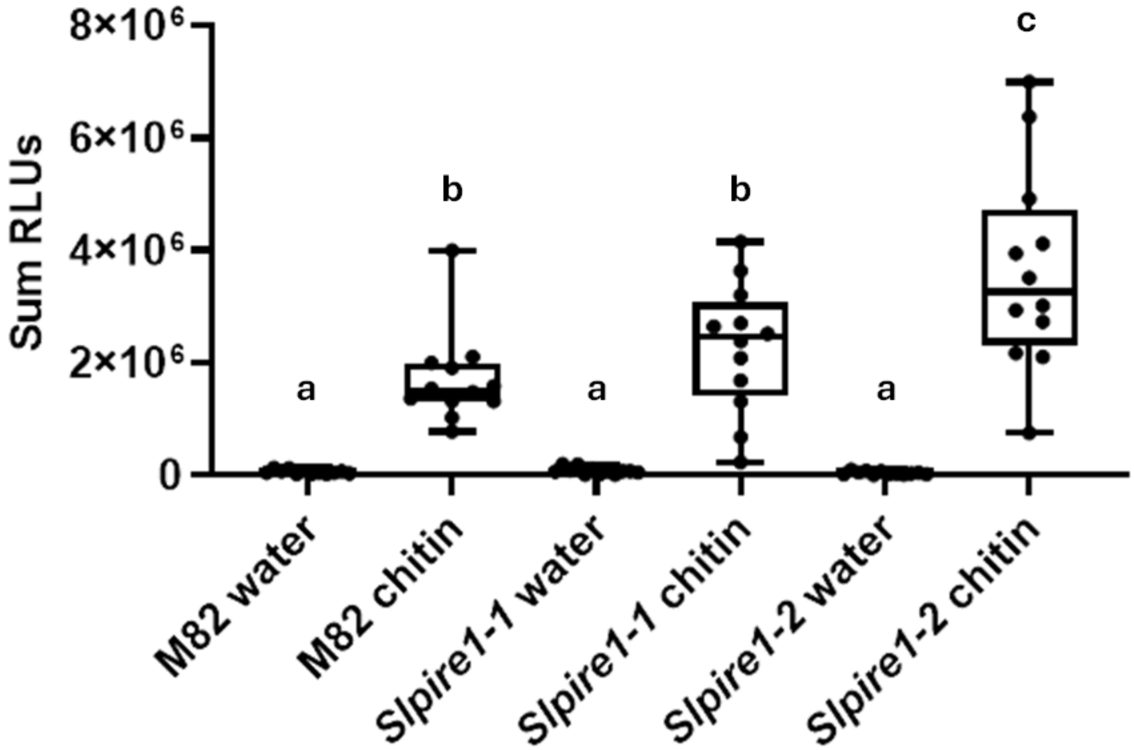

Supplementary Figure S6. ROS production after induction with chitin.

Cumulative Sum RLUs of ROS burst after chitin induction. *S. lycopersicum* M82, *Slpire1-1,* and *Slpire1-2* leaf disks were collected and treated with 10μM chitin to induce ROS production over 60 minutes. Results display the sum of RLUs produced by the leaf disks*,* RLU = relative light units. Four biological replicates per experiment (N=4) Whiskers represent the maximum and minimum values. Water controls display no ROS burst. Statistical differences were determined by ANOVA with post-hoc Tukey test (p value < 0.0001). Experiments were repeated three times.

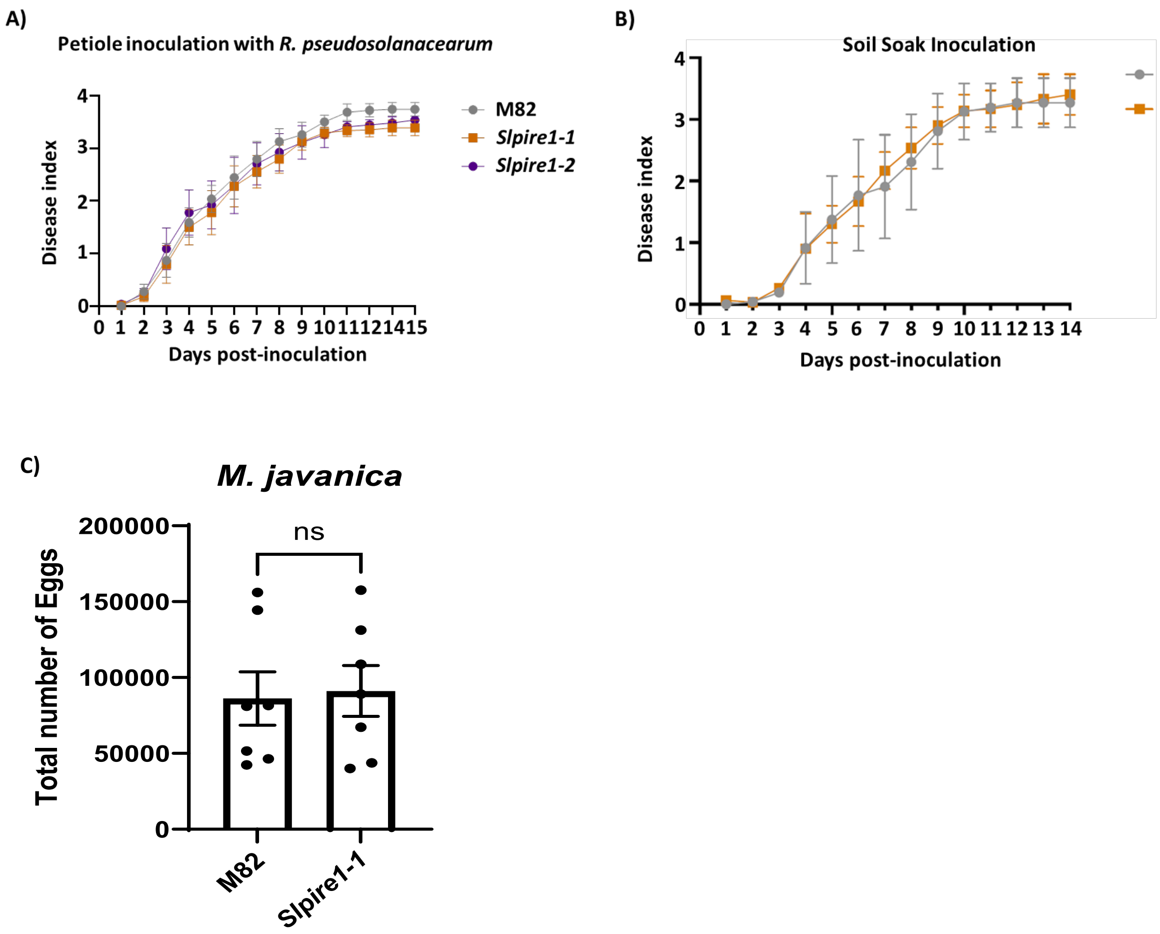

Supplementary Figure S7. Disease measurements for *R. pseudosolanacearum* GMI1000 strains and *M. javanica* strains on M82 (wild type) and *Slpire1*. A) The x-axis represents the days post-inoculation, and the y-axis represents the disease index. Error bars correspond to the standard deviation of the Mean (SEM). This experiment was repeated in three biological replicates, and in each replicate N=18 plants were inoculated at the petiole level. **B)** Representative graph of egg counts on infected roots. 4-week-old plants were infected with 500 J2 stage nematodes. Nematode egg counts were performed 7 weeks post infection. Error bars correspond to the standard deviation of the Mean

(SEM). This experiment was repeated 2 times with 7 biological replicates per experiment N=7 plants were inoculated. Statistical differences were detected by t-test, alpha = 0.05, ns = not significant.
